# Associations between Ectomycorrhizal Fungi and Bacterial Needle Endophytes in *Pinus radiata*: Implications for Biotic Selection of Microbial Communities

**DOI:** 10.3389/fmicb.2016.00399

**Published:** 2016-03-31

**Authors:** Megan A. Rúa, Emily C. Wilson, Sarah Steele, Arielle R. Munters, Jason D. Hoeksema, Anna C. Frank

**Affiliations:** ^1^Department of Biology, University of Mississippi, OxfordMS, USA; ^2^National Institute for Mathematical and Biological Synthesis, University of Tennessee, KnoxvilleTN, USA; ^3^Life and Environmental Sciences and Sierra Nevada Research Institute, School of Natural Sciences, University of California, Merced, MercedCA, USA

**Keywords:** 16S rRNA, bacterial endophytes, conifers, exploration types, foraging traits, *Gammaproteobacteria*, soil texture

## Abstract

Studies of the ecological and evolutionary relationships between plants and their associated microbes have long been focused on single microbes, or single microbial guilds, but in reality, plants associate with a diverse array of microbes from a varied set of guilds. As such, multitrophic interactions among plant-associated microbes from multiple guilds represent an area of developing research, and can reveal how complex microbial communities are structured around plants. Interactions between coniferous plants and their associated microbes provide a good model system for such studies, as conifers host a suite of microorganisms including mutualistic ectomycorrhizal (ECM) fungi and foliar bacterial endophytes. To investigate the potential role ECM fungi play in structuring foliar bacterial endophyte communities, we sampled three isolated, native populations of Monterey pine (*Pinus radiata*), and used constrained analysis of principal coordinates to relate the community matrices of the ECM fungi and bacterial endophytes. Our results suggest that ECM fungi may be important factors for explaining variation in bacterial endophyte communities but this effect is influenced by population and environmental characteristics, emphasizing the potential importance of other factors — biotic or abiotic — in determining the composition of bacterial communities. We also classified ECM fungi into categories based on known fungal traits associated with substrate exploration and nutrient mobilization strategies since variation in these traits allows the fungi to acquire nutrients across a wide range of abiotic conditions and may influence the outcome of multi-species interactions. Across populations and environmental factors, none of the traits associated with fungal foraging strategy types significantly structured bacterial assemblages, suggesting these ECM fungal traits are not important for understanding endophyte-ECM interactions. Overall, our results suggest that both biotic species interactions and environmental filtering are important for structuring microbial communities but emphasize the need for more research into these interactions.

## Introduction

All plants associate with a diverse array of microorganisms throughout their tissues, but microbes associated with above- and belowground portions of plants are typically studied separately. However, a growing body of research indicates that above- and belowground multitrophic interactions have important implications for both plant community structure and ecosystem function (Wardle et al., 2004; Van Der Putten, 2009; Wagg et al., 2011; Rúa et al., 2013). Such interactions are intrinsically linked for plant-associated microbes since many microbes may have physiological effects on plant performance that span the entirety of the plant (as opposed to localized to the direct area they occupy); therefore, plant-associated microbes may play an important role in the structure and dynamics of one another, even if they occupy spaces within the tree that are a significant distance from one another. Furthermore, our knowledge concerning the ecology and interactions of microbes in plants is likely biased because studies of plant–microbial interactions are commonly based on controlled, optimized conditions for growth of host plants and seldom based on variable, field-realistic conditions. In addition, the degree to which non-wild and wild systems are comparable for plant–microbe interactions remains unclear as there are major differences in both phylogenetic diversity and species composition of plant hosts between agroecosystems and the associated forest regions (Griffin and Carson, 2015).

Coniferous plants and their associated microbes provide a good model system for exploring the interaction of above- and belowground plant-associated microbes as conifers interact with a suite of microorganisms including mutualistic mycorrhizal fungi (Smith and Read, 2008), pathogenic eukaryotic microbes (Fogel, 1988), and foliar bacterial and fungal endophytes (Carroll, 1988; Pirttilä et al., 2005; Arnold et al., 2007; Carrell and Frank, 2014, 2015; Oono et al., 2014). The relationships between conifers and their microbes may be particularly important since conifers grow in impoverished acidic soils at both mid and high latitudes, potentially facilitated by their relationship with various microbes (Axelrod, 1986; Lepage et al., 1997; Richardson, 2000; Carrell and Frank, 2014).

The nutritional mutualism between mycorrhizal fungi and plants is a key component of a conifer’s ecological niche, as the two organisms are believed to have co-diversified approximately 200 million years ago and now form functionally obligate associations (Smith and Read, 2008; Tedersoo et al., 2010). These relationships are characterized by the exchange of carbon (C) in the form of carbohydrates from the plant for mineral nutrients such as nitrogen (N), phosphorus (P), and micronutrients supplied by the fungi (Smith and Read, 2008). Conifers associate primarily with ectomycorrhizal (ECM) fungi, an extremely diverse group that contains more than 5,000 described species from the fungal order Agaricales alone (Ryberg and Matheny, 2012). ECM fungi are particularly important for conifers as they improve plant growth and confer resistance against biotic and abiotic stresses (Smith and Read, 2008). The degree to which ECM fungi are able to confer such resistance is likely related to their ability to forage the litter and mineral layers for nutrients (Agerer, 2001; Selosse et al., 2006; Anderson and Cairney, 2007; Hobbie and Agerer, 2010). The extramatrical mycelium, which extends out into the soil from ECM root tips and facilitates nutrient uptake and transport, has evolved distinct characteristics in different taxa of ECM fungi related to their ability to acquire nutrients. These characteristics include mycelial growth pattern, extent of biomass accumulation, hydrophobicity of the hyphae, and the presence or absence of rhizomorphs (linear aggregations of parallel-oriented hyphae) are used to classify fungi into exploration types. These exploration types reflect differences among taxa in exploration and nutrient mobilization strategy such that variation in these traits allows the fungi to acquire nutrients across a wide range of abiotic conditions (Agerer, 2001; Hobbie and Agerer, 2010; Lilleskov et al., 2011). For example, medium-distance fringe and long-distance exploration types have the potential to explore recalcitrant nutrient pools while species with contact, short, and medium-distance smooth exploration types tend to be associated with labile nutrient soil pools (Lilleskov et al., 2002; Hobbie and Agerer, 2010). While these characteristics primarily provide resistance to abiotic stresses, they are also likely to confer resistance to biotic stresses, particularly by increasing a plant’s overall tolerance to stress. Using a trait-based approach to ECM fungal ecology has been an important tool for studying the effect of ECM on ecosystem processes such as nitrogen and carbon cycling (Krause et al., 2014; Aguilar-Trigueros et al., 2015), but it may also provide an important framework to link ECM diversity to species interactions with other microorganisms.

Although belowground plant-associated microbial communities have received extensive attention (Bever, 2003; Van Der Heijden et al., 2008; van der Putten et al., 2013), their aboveground counterparts remain an area of developing research. The study of endophytes (microorganisms that spend at least part of their life cycle inside plants), has traditionally focused on fungi that live inside the aboveground portion of plants (Schardl and Phillips, 1997; Six et al., 2006). In contrast, knowledge of the role of aboveground bacterial endophytes in plant growth, plant nutrient uptake, and protection against biotic and abiotic stress in wild plants is growing (Chanway, 1996).

The needles of conifers are heavily colonized by fungal endophytes (Pirttilä and Wäli, 2009), and the number of infections typically increases with needle age and varies with position such that needles and buds tend to differ in their fungal endophyte communities. Less is known about the endophytic bacteria in conifer foliage and their ecological interactions with plants. Recent 16S rRNA surveys of the endophytic community in high elevation pines suggest that these trees select specific taxa that recur within and between *Pinus* species growing in the subalpine environment (Carrell and Frank, 2014). The foliar endophyte communities of coast redwood and giant sequoia are more variable and diverse, but clearly select for specific bacterial lineages that are also shared with other wild plants (and lichens) (Carrell and Frank, 2015). Indeed, conifer–bacteria interactions can be very intimate, as illustrated by the plant growth promoting bacterial endophyte *Methylobacterium extorquens* DSM 1360, which occupies a niche within the plant cell in association with the nucleus (Koskimäki et al., 2015).

Evidence from agricultural crop species and *Arabidopsis thaliana* suggests that bacterial endophyte communities are structured based on C usage (Ji and Wilson, 2002; Innerebner et al., 2011), but structuring factors are less understood in wild plants. Additional research suggests that foliar bacterial endophytes may serve an important role as N_2_ fixers for a wide taxonomic range of plants including conifers (James, 2000; Carrell and Frank, 2014; Chanway et al., 2014), and incorporation of fixed N by the plant host has been demonstrated in several systems (Sevilla et al., 2001; Pankievicz et al., 2015). Consequently, environmental factors that alter plant nutrient acquisition, including nutritional relationships with other microbial symbionts such as ECM fungi, may be important for structuring bacterial endophyte communities.

While studies examining the interaction of ECM fungi and bacterial endophytes are limited, recent laboratory work indicates that the outcome of the interaction between foliar bacterial endophytes and ECM fungi is dependent upon the identity of the fungus. In a highly controlled study examining the interaction of an ECM fungus (*Pisolithus tinctorius* or *Suillus variegatus*) and the bacterial pine endophyte *M. extorquens* DSM 1360 on the growth of Scots pine (*Pinus sylvestris*) seedlings, identity of the ECM fungi was important for determining the overall effect of the interaction. Specifically, seedlings inoculated with *P. tinctorius* and *M. extorquens*, had greater overall biomass than those grown without microbes or with only one microbe (Pohjanen et al., 2014). In contrast, seedlings inoculated with *S. variegatus* and *M. extorquens* were larger than the non-microbe control but did not differ in overall biomass from those inoculated with only a single microbe (Pohjanen et al., 2014). This study, while limited to one bacterial species, suggests fungal identity is an important factor in determining the outcome of ECM-bacterial endophyte interactions in pines and suggests belowground ECM fungal communities have the potential to structure aboveground bacterial endophytes; however, in order to more fully understand the interaction of ECM fungi and bacterial endophytes, research that evaluates diverse, natural ECM fungal communities and bacterial endophytes in adult trees is necessary.

In our study, we investigate the extent to which the belowground ECM fungal community of a conifer is associated with its aboveground foliar bacterial endophyte communities, and whether host population and environmental characteristics alter this relationship. We focused on Monterey Pine (*Pinus radiata* D. Don), a locally dominant conifer with a native range that consists of small isolated populations spanning a broad latitudinal gradient along the west coast of California and Mexico (Grotkopp et al., 2004). Previous work with *P. radiata* indicates that different populations harbor different ECM fungal communities, at least in part due to genetic differences among host populations (Hoeksema and Thompson, 2007; Hoeksema et al., 2012) but to our knowledge, their foliar bacterial endophytes have never been characterized. Via molecular methods, we identified ECM fungal communities (ITS-1F and ITS4) and foliar bacterial endophytes (16S rRNA) for three isolated populations of *P. radiata* in California (Año Nuevo, Monterey, and Cambria). We then used multivariate modeling approaches to explore the extent to which population and environmental factors shaped ECM and endophyte communities independently, how the microbe communities related to one another, and whether these patterns were influenced by either population or environmental characteristics.

## Materials and Methods

### Description of Field Site and Field Sampling Methods

Soil and foliage were collected from stands in three native Monterey pine populations in mainland California: Swanton Pacific Ranch, Año Nuevo, CA, USA (37° 3.9′ N, 122° 14.5′ W), Point Lobos State Natural Reserve, Monterey, CA, USA (36° 30.9′ N, 121° 56.6′ W) and at the Kenneth S. Norris Rancho Marino Reserve, Cambria, CA, USA (35° 32.2′ N, 121° 04.7′ W). Monterey pine was the dominant ECM tree species at all three sampling sites, although occasional Douglas-firs (*Pseudotsuga menziesii*) were observed at Año Nuevo, and occasional coast live oaks (*Quercus agrifolia*) were observed at both Cambria and Año Nuevo. In addition, knobcone pine (*Pinus attenuata*) is known to occur in the Año Nuevo population and to hybridize with Monterey pine, but no knobcone pines were observed in the vicinity of our specific sampling site.

Between February 21–23, 2014 we sampled five haphazardly chosen trees at each site that were similar in size as determined by diameter at breast height (DBH). The distance between the two farthest spaced trees at each site varied from 0.14 km (at Monterey and Cambria) to 1.67 km (at Año Nuevo). Power analyses that were undertaken prior to sampling suggested that five trees per site would be adequate to detect the patterns we were interested in exploring. Soil was obtained by collecting four cores per tree per site (total 20 cores), each 10 cm in diameter and 20 cm deep (excluding litter) from four locations chosen in each cardinal direction within 1 m around a focal tree to obtain a representative sample of each tree. Soil samples were stored in insulated coolers, overnighted to the laboratory at the University of Mississippi, stored at 4°C, and processed within 17 days of collection. All root samples and surrounding soil were kept intact until processing.

Needle and bud tissue samples were collected from each of the five trees per site. Five to ten small needle bundles were collected from each tree, at the North, South, East, and West sides of the tree to obtain a representative sampling at approximately 2 m height. Buds were collected when present and pooled by site. Samples were collected with sterile gloves and blades, and large sterile sample bags were used to minimize tissue damage. Samples were stored in cool conditions for transport back to the lab for surface sterilization within 1 week of sampling.

### Bacterial Sequencing and OTU Generation

#### Sample Sterilization

All foliage samples were processed at UC Merced. Foliage was sterilized by submersion in 30% hydrogen peroxide for 1 min followed by 10, 1 min shaking rinses in ultrapure water and stored at -20°C. Sterility was confirmed by negative PCR amplification of the final rinse. Negative PCR amplification of the final rinse suggests sterility; however, microscopy would be necessary to verify sterility. Thus, it is possible that some of the bacterial taxa we observe are epiphytes.

#### DNA Extraction for Bacterial Analysis

Foliage samples were ground to a fine powder with a sterile mortar and pestle on a liquid nitrogen filled-base. DNA was extracted using a CTAB extraction method as described previously (Carrell and Frank, 2014). Briefly, 800 μL of CTAB solution (1 mL CTAB buffer, 0.04 g of polyvinylpyrrolidone, 5 μL of 2-mercaptoethanol) was added to 0.6 g of tissue, incubated for 2 h at 60°C, and homogenized with sterile glass beads for 3 min. Proteins were removed with the addition of an equal volume of 24:1 molecular grade chloroform/isoamyl alcohol, centrifuged for 10 min at 16 rcf, and the top aqueous phase was placed in a sterile tube. Nucleic acids were precipitated with the addition of 1/10 volume of cold 3 M sodium acetate and 1/1 volume cold isopropanol, placed in a -20°C freezer for 12 h, followed by centrifugation for 30 min at 16 rcf to pellet the DNA. The supernatant was removed, 700 μL of 70% ethanol was added, then the tube was shaken to dislodge and rinse the pellet, followed by centrifugation for 10 min. The ethanol was then removed and the pellet was air dried in sterile conditions. The air-dried pellet was re-suspended with 30 μL of DNA suspension buffer (1.0 M Tris-HCL, 0.1 M EDTA) and stored at -20°C.

#### DNA Amplification for Bacterial Analysis

The 16S rRNA genes of endophytic bacteria were amplified using nested PCR to reduce the occurrence of plastid sequences and improve consistency. Plant DNA amplification was suppressed with the primer pair 16S 799f (AACMGGATTAGATACCCKG) and 16S 1492r (TACGGHTACCTTGTTACGACT) in the first PCR reaction (PCR1). Amplification with 16S 799f and 16S 1492r result in mitochondrial amplicon of about 1000 bp and bacterial amplicon of about 750 bp (Chelius and Triplett, 2001). In the final round of PCR (PCR2), an appropriate amplicon length for Illumina sequences was achieved from PCR1 amplicons with the Illumina adapted, Golay-barcoded primer pair 799f and 1115r (AGGGTTGCGCTCGTTG), an optimized primer set for phylogenetic analysis of short reads (Redford et al., 2010) reduced primer bias by decreasing the number of cycles (Jiao et al., 2006) with the following thermocycle profile used for PCR1 and PCR2: one cycle of 3 min at 95°C; 20 cycles of 40 s at 95°C, 40 s at 50°C, 1.5 min at 72°C; and a final 10 min of elongation at 72°C. The 50 μL PCR1 reaction contained 5 μL of DNA extract, 20 μL 5 PRIME Hot Master Mix (5 PRIME, Inc.), 0.5 μg/μl Bovine Serum Albumin (Thermo Scientific), 21.5 μL PCR grade water (Fisher BioReagents), and 0.2 μM of forward and reverse primers. The 25 μL PCR2 reaction contained 3 μL of PCR1 product, 10 μL 5 PRIME Hot Master Mix, 0.5 μg/μL Bovine Serum Albumin (Thermo Scientific), 8.75 μL PCR grade water (Fisher BioReagents), and 0.2 μM of forward and reverse primers. PCR2 amplicons were then cleaned, pooled, and gel extracted (QIAquick Gel Extraction Kit) to ensure selection of the correct band size and to remove most mitochondrial products. Pooled samples were then submitted for Illumina sequencing at the University of California, Davis Genome Center.

#### OTU Generation and Classification for Bacterial Analysis

Sequences were analyzed and processed using the QIIME (1.8.0) package (Caporaso et al., 2010b), but with the UPARSE method for clustering operational taxonomic units (OTUs) (Edgar, 2013). Forward and reverse paired-end reads were joined with fastq-join, with the barcode filtered from the dataset if the forward and reverse read did not overlap (Aronesty, 2011). Joined pair-end reads were quality filtered with QIIME defaults settings (maximum number of consecutive low quality base calls of three bases; minimum number of consecutive high quality base calls as a fraction of the input read length of 0.5 total read length; maximum unacceptable Phred quality score of 3; no N characters) which have been found to sufficient for community analysis (Bokulich et al., 2013). The remaining sequences were clustered into OTUs at the 97% level. Representative sequences were aligned using PyNAST (Caporaso et al., 2010a) against the Greengenes core set (DeSantis et al., 2006). Taxonomic assignments were made using uclust (Edgar, 2010) with the Greengenes representative set of sequences as reference. Sequences classified as “Chloroplast,” “Mitochondria” or “Unassigned” were removed. An approximately maximum-likelihood tree was constructed from an alignment of representative sequences, using FastTree (Price et al., 2009).

### Fungal Sequencing and OTU Generation

#### Laboratory Processing of Root Samples and Morphotyping

Roots were hand washed over a 2 mm sieve to remove rhizosphere soil. All roots within a sample were pooled and the root-tip region encompassed by the fungal hartig net of the ECM fungi was assessed using a dissecting microscope. The number of root tips with viable ECM fungal colonization was counted, and each root tip was classified into a morphotype based on morphological distinctions such as color, texture, branching patterns, and emanating hyphae or rhizomorphs. Two root tips per morphotype observed in each sample were removed for identification via Sanger sequencing. All laboratory processing of root samples and morphotyping occurred within 17 days of collection.

#### DNA Extraction and Sequencing

After morphotyping, DNA was immediately extracted from root tips from each sample using components of a Sigma Extract-N-Amp extraction kit (Sigma–Aldrich, St. Louis, MO, USA). Ten microliter of the Sigma Extraction Buffer was added to each root tip, which was heated to 65°C for 10 min, 95°C for 10 min, and then received 30 μL of Sigma Neutralization Solution and 60 μL PCR-grade water. Samples were then stored at -20°C for approximately 1 month.

To facilitate Sanger sequencing of ECM fungal species colonizing root tips, the Internal Transcribed Spacer (ITS) region of the fungal nuclear genome was amplified using the fungal-specific forward and reverse primers, ITS1F and ITS4 (Gardes and Bruns, 1993). Amplification was achieved according to the protocol outlined in Rúa et al. (2015) and success was checked on a 1% agarose gel with SYBR^®^ Safe DNA gel stain (Molecular Probes, Eugene, OR, USA). Approximately 10% of samples did not amplify with the default settings. The raw DNA for these samples was diluted to 1% DNA (1 μl DNA +99 μl sterile PCR-grade water) and amplification reactions were repeated using the same methods as the default except that the number of cycles for denaturation, annealing, and extension was increased from 35 cycles to 40 cycles. Approximately 5% of dilutions did not amplify with these settings so amplification was repeated with the same methods as the previous dilutions and the annealing temperature was lowered from 52°C to 51°C.

Excess primer and unincorporated nucleotides were removed enzymatically using the Exonuclease I (ExoI) and Antarctic Phosphotase (AP) enzymes (New England BioLabs, Inc., Ipswich, MA, USA) with the following procedure: 0.5 μl ExoI, 0.5 μl AP, and 4.5 μl sterile PCR-grade water were added to 5 μl of the PCR product and incubated at 37°C for 45 min, then 80°C for 20 min, and finally 4°C for at least 5 min.

Sequencing was performed using the forward primer ITS5 (Gardes and Bruns, 1993) and the Big Dye Terminator Sequencing Kit (v3.1, Invitrogen Corp.). Each Big Dye reaction contained 0.4 μL Big Dye Reaction Premix, 1.8 μL Big Dye 5 X sequencing buffer, 0.5 μL of the forward primer at 10 μM concentration, 6.3 μL of PCR-grade water, and 1 μL of the cleaned PCR product. Amplification conditions were 96°C for 1 min; followed by 35 cycles of 95°C for 30 s, 50°C for 20 s, and 60°C for 4 min. Reactions were dried and shipped overnight to the DNA Lab at Arizona State University, in Tempe, AZ, USA, where the Big Dye reactions were purified and read on an Applied Bioscience 3730 capillary genetic analyzer. We chose to sequence the fungal samples in one direction as this approach provides plenty of high-quality sequences of sufficient length for OTU assembly and taxonomic matching while keeping costs low. The main effect of only sequencing in one direction is to modestly lower the quality of some sequences, but this should not bias the results in a meaningful way, except perhaps to increase the number of singlet OTUs.

#### Sequence Assignment

The fungal DNA sequences obtained were edited manually in Geneious software (Biomatter Ltd.), correcting ambiguous bases associated with dye blobs and elsewhere when possible. All sequences with >3% ambiguous bases or <200 base pairs long were deleted. Remaining sequences were subjected to OTU assembly (at 97% similarity) using CAP3 software (Huang and Madan, 1999) on the University of Alaska, Fairbanks (UAF) Life Science Informatics server, using default settings except the following: maximum overhang percent length = 60, match score factor = 6, overlap percent identity cut-off = 97, clipping range = 6, as described previously in (Rúa et al., 2015). Grouping homologous sequences that are >97% similar as a specific OTU is a conservative approach employed by previous studies (Izzo et al., 2005; O’Brien et al., 2005; Smith et al., 2007) and assumes a 0.2–1.2% error rate produced by PCR and unidirectional sequencing, as well as ∼1.5% divergence of the ITS region that may occur within some species at small spatial scales (Horton, 2002).

Consensus fungal sequences from each OTU were checked using BLAST (Altschul et al., 1990; nucleotide) searches on the International Nucleotide Sequence Database (INSD) and the User-Friendly Nordic ITS Ectomycorrhizal (UNITE) database (Kõljalg et al., 2013) to obtain best matches for taxonomic affiliation of OTUs. The ultimate decision on the best match to a sequence was based on both similarity and length of the match. Sequences >97% similar in composition to database sequences from named, cultured fungi were considered the same OTU (hereafter, ‘species’). Sequences with matches showing 94–97% similarity to a database sequence with an assigned species epithet, or matching a sequence identified only to genus were assigned into the respective genus and assigned a number (e.g., *Russula* 1). Similarly, those matches in the database <94%, but greater than 90% were assigned to the appropriate taxonomic family. Any matches <90% similar to database sequences were left out of the analyses. If sequence matches among the two sequence repositories showed equal affinity or similarity to multiple genera within a family, priority was given to the vouchered specimens residing on the UNITE or curated fungal ITS databases. Any species known to be strictly pathogenic was eliminated from the data set.

When fungal OTUs could be identified to species, fungal traits associated with foraging strategy, foraging distance, rhizomorph formation, and hydrophobicity (Agerer, 2001), were assigned using the Determination of EctoMYcorrhiza database (DEEMY, http://www.deemy.de). Since foraging related functional traits are typically conserved at the genus level (Agerer, 2006), when no species-level matches were available in DEEMY, entries for congeners associated with *Pinus* were surveyed. Consensus trait values were assigned if 90% of entries agreed which allowed for data that could only be identified to genus to be incorporated (sensu Moeller et al., 2014).

### Soil Characterization

Soil texture (% sand, % silt, % clay) was characterized for each soil sample (Lamotte soil texture test kit). Soil water content was determined as the ratio of the initial soil weight to the soil weight after being dried in a drying oven at 65°C for 72 h.

### Statistical Analyses

Before analyses fungal and bacterial data were pooled by tree, abundance of each ECM fungal species were relativized by the total abundance observed in the data set (McCune and Grace, 2002) in order to put all species abundances on the same relative scale. Bacterial OTUs were rarified to an even sampling depth of 7364 sequences (the smallest amount of sequences for any sample) and OTUs with greater than 100 observations were selected for further downstream analyses (R Core Team, 2015). After demultiplexing, quality control, OTU clustering, taxonomic identification and rarefaction using QIIME, version 1.8.0 and UPARSE as described above, the bacterial OTU table was exported into R using the *phyloseq* package for further analyses (McMurdie and Holmes, 2013). All subsequent analyses were done with R statistical software, version 3.1.3.

To assess variation in environmental factors due to population (site), each environmental factor (% silt, % sand, % clay, soil water content) was subjected to a linear model using the *lm* function from the *stats* package (R Core Team, 2015). All environmental factors were tested for normality and found to possess normal distributions. Differences between sites were determined with a Tukey’s multiple comparison test using the *HSD.test* function from the *agricolae* package (de Mendiburu, 2014). Environmental variables were further subject to correlation analysis using the *cor* function of the *stats* package (R Core Team, 2015) and analyzed using principal components analysis (PCA) after centering and standardization of the variables using the *prcomp* function from the *stats* package (R Core Team, 2015). Visualizations for these analyses were created by adapting the *ggbiplot* function from the *ggbiplot* package (Vu, 2011) and using the *ggplot2* package (Wickham, 2009). Percent silt and % sand were found to be correlated (**Table 1**: -0.7985, *p* < 0.0001) but when combined into a single metric, results were not qualitatively different then when left apart (see Supplementary Material). We briefly mention in the text the results obtained with the combined variable.

**Table 1:** Correlation coefficients and associated *p*-values for environmental variables.

	Soil water content		% Clay		% Sand		% Silt
Soil water content	1.000						
% Clay	0.2950	0.0012^∗∗^	1.000				
% Sand	0.2409	0.0086^∗∗^	-0.2815	0.0020^∗∗^	1.000		
% Silt	-0.4200	<0.0001^∗∗∗^	-0.3529	<0.0001^∗∗∗^	-0.7985	<0.0001^∗∗∗^	1.000


*Significance codes: 0 < ^∗∗∗^ < 0.001 < ^∗∗^ < 0.01 < ^∗^ < 0.05 < . < 0.1. Pearson’s r rho rank correlation coefficients for all possible pairs of environmental variables and the associated asymptotic p-values. If the correlation coefficient is close to 1, then the variables are positively linearly related. If the correlation coefficient is close to -1, then the variables are negatively linearly related.*

#### Community Analysis

Alpha diversity (the diversity within a single tree) was estimated using the Chao1 index which measures species richness, and the Shannon diversity index which integrates richness and evenness. Both indices were calculated via functions in the *vegan* package (*estimateR* to calculate the Chao1 index and *diversity* to calculate the Shannon index; Oksanen et al., 2015). Variation in richness and diversity among locations (beta diversity) was assessed using linear models with the *lm* function and population as the predictor variable. To determine the frequency of different ECM fungal foraging strategies by population, we used Pearson’s chi-squared tests from the *xtabs* function in the *stats* package (R Core Team, 2015). To determine if the distribution of traits associated with ECM fungal foraging strategy (response variable) changes as a result of the environmental characteristics (predictor variable), we used logistic regression via the *glm* function with poisson family and log link in the *stats* package (R Core Team, 2015).

To assess the sources of variation in the microbial community matrices due to population, environmental factors, fungal foraging strategy (ECM only), and tissue type (bacteria only) we used permutational manova based on 999 permutations and the Bray–Curtis method for calculating dissimilarity indices via the *adonis* function in the *vegan* package (Oksanen et al., 2015). Visualizations for this analysis were created using NMDS plots and the Bray–Curtis method via the *metaMDS* function in the *vegan* package and the *ggplot2* package (Wickham, 2009). In order to determine if any fungal or bacterial species were driving patterns due to population or tissue type (bacteria), we calculated correlation indices with the *mulitpatt* function from the *indicspecies* package (De Cáceres and Legendre, 2009) and corrected the resulting phi coefficient for the fact that some species may be present at more sites then others (De Cáceres et al., 2010). Analyses were run with 1000 permutations. Because the environmental variables are continuous, in order to examine the role environmental variables played in influencing the abundance of microbes, we conducted correlation analyses between individual OTU abundances and single environmental variables with the *cor* function of the *stats* package (R Core Team, 2015).

We used constrained analysis of principal coordinates (CAP) which allows for the use of non-Euclidean dissimilarity indices, such as Jaccard or Bray–Curtis distance, to relate the community matrices of the ECM fungi and bacterial endophytes. First, matrices were restricted to those microbes that were identified from multiple trees within a population and from all populations (67 bacterial species and 5 fungal species). Because our bacterial species matrix was significantly larger than our fungal species matrix, we were able to explore the extent to which fungal species were associated with bacterial species, but could not explore the reverse question regarding the extent to which bacterial species can structure the fungal communities. To do this we utilized the *capscale* function from the *vegan* package to determine whether fungal species influenced the bacterial species community (Oksanen et al., 2015). This function performs a constrained ordination describing variation in the bacterial species community composition along defined axes – the composite variables representing fungal community composition – then proceeds to explain the remaining variation via unconstrained ordination (Oksanen et al., 2015). The resulting output was then subjected to Type I ANOVA with 999 permutations to determine which ECM fungal species were influencing the bacterial community. Subsequent models were run using the ‘condition’ option of *capscale* which removes variation from the designated variable from the ordination before constraining for other variables (a ‘partial’ CAP). Separate models were run that conditioned for population or environmental variables. Finally, analyses were repeated using ECM fungal traits (rather than ECM species identity) to examine their influence in shaping the bacterial endophyte community. All analyses were initially run using Bray-Curtis distance but were repeated using Jaccard distances. Since analyses did not differ, we present analyses with Bray–Curtis distance here. Visualizations were created by modifying the *autoplot* function in *ggvegan* (Simpson, 2015).

## Results

### Environmental Characteristics

Linear models indicate the three *P. radiata* populations differed significantly in several environmental factors (**Supplementary Figure S1**) including soil water content (*F*_2,15_ = 87.97, *p* < 0.0001) and all three measures of soil texture (% silt: *F*_2,15_ = 163.8, *p* < 0.0001, % sand: *F*_2,15_ = 39.96, *p* < 0.0001, % clay: *F*_2,15_ = 104.1, *p* < 0.0001). Specifically, % silt is highest for Monterey, % clay is highest for Cambria, and Año Nuevo has higher % sand and soil water content (**Supplementary Figure S1**). The correlation structure within the set of environmental variables was visualized using PCA (**Supplementary Figure S2**). The first two axes describe 83.8% of the total variation. Population was clearly related to the environmental variables. There was a negative correlation between % silt and % sand (-0.7984) but while the other environmental characteristics were significantly correlated (**Table 1**), no other patterns were strong. When % silt and % sand were combined into a single metric, Año Nuevo has higher % sand: % silt and soil water content [**Supplementary Figure S1**, % sand: %silt (*F*_2,15_ = 39.04, *p* < 0.0001)].

### Bacterial Endophytes

#### Overall Community Description

A total of 83 distinct bacterial OTUs were recovered from the 15 needle and three bud samples. These sequence data have been submitted to the GenBank databases under BioProject accession number PRJNA303051. Rarefaction plots saturated, indicating that we did not under-sample the bacterial communities at the 97% OTU level (**Supplementary Figure S3**). Overall our samples consisted of 20 bacterial families. There were three families (Bdellovibrionaceae, Methylobacteriaceae, and Salinisphaeraceae) unique to buds (not found in the needles).

**FIGURE 1:**
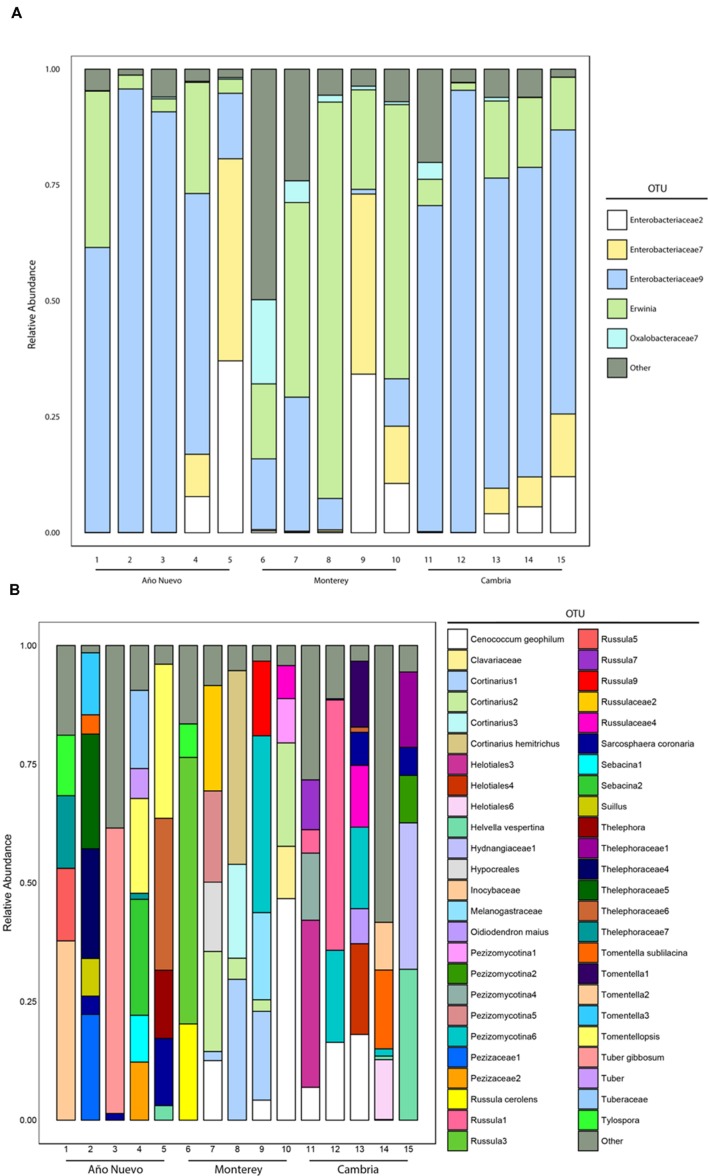
**Relative abundances of various major bacterial **(A)** and fungal **(B)** taxa recovered from Año Nuevo, Monterey, and Cambria, CA, USA.** Relative abundance of families was calculated as the proportion of sequences belonging to a particular lineage of all 16S rRNA (bacteria) and ITS (fungi) gene sequences recovered from each site. Sequences were pooled at the level of the tree. Only those OTUs which represent >90% of the total tips are identified separately via color while the remaining OTUs were grouped into an ‘Other’ category for visualization.

Our samples were dominated by Proteobacteria (94% of total sequences), the majority of which belonged to the class Gammaproteobacteria (88% of total sequences). No other phylum was present above 1%. Within the Proteobacteria, other classes were present in low relative abundance (e.g., Alphaproteobacteria 1.6%, and Betaproteobacteria 3.9% of total sequences). The dominance of Gammaproteobacteria reflects the dominance of a few specific OTUs in most of our samples (**Figure 1A**). The most common OTU in our dataset, Enterobacteriaceae9, belonged to the Enterobacteriaceae, and made up on average 70, 12, and 53% of the sequences from Cambria, Monterey, and Año Nuevo samples, respectively. This OTU shares 100% identity to sequences from *Rahnella*, *Serratia*, and *Ewingella* isolates and clones from various plant tissues. The second most common OTU (*Erwinia*) was present at a higher relative abundance in the Monterey samples (42%) than the samples from Cambria (11%) and Ano Nuevo (11%). Two other OTUs (Enterobacteriaceae7 and Enterobacteriaceae2) belonging to the Enterobacteriaceae were also present at high relative abundance in many samples (**Figure 1A**).

#### Community Structure as a Function of Population

Alpha diversity (the diversity of needle microbiota within a single tree) was estimated by Chao1 and Shannon indices (**Supplementary Figures S4A–C**). No difference in alpha diversity was found among locations for either the Chao1 index (*F*_2,12_ = 2.129, *p* = 0.1617) or the Shannon index (*F*_2,12_ = 2.836, *p* = 0.098).

**FIGURE 2:**
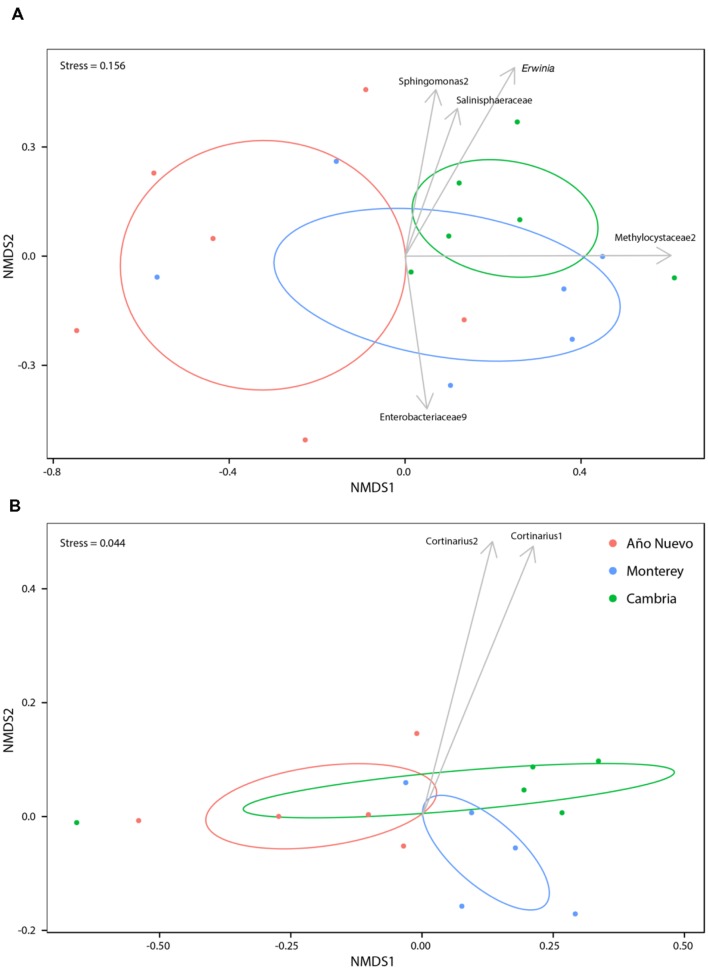
**Non-metric multidimensional scaling ordinations for bacteria **(A)** and fungi **(B)** communities as a function of collection site.** Ellipses indicate 95% confidence intervals for samples collected from Año Nuevo (salmon), Monterey (green), and Cambria (blue). Arrows specify significant indicator species associated with particular sites.

Bacterial community abundances were sorted into an ordination plot according to community similarity (**Figure 2A**). There was significant clustering based on population (*R*^2^ = 0.35, *p* = 0.006). Four OTUs were significantly associated with Monterey while a single OTU was significantly associated with both Cambria and Año Nuevo. *Sphingomonas2* (stat = 0.719, *p* = 0.006), *Erwinia* (stat = 0.680, *p* = 0.003), Methylocystaceae2 (stat = 0.513, *p* = 0.023), and Salinisphaeraceae (stat = 0.383, *p* = 0.038) are strongly and significantly associated with Monterey while Enterobacteriaceae9 (stat = 0.716, *p* = 0.006) is strongly and significantly associated with Cambria and Año Nuevo (**Figure 2A**).

#### Community Structure as a Function of Environmental Characteristics

Environmental characteristics were important variables for shaping bacterial alpha diversity; however, specific results differed by indices (**Supplementary Figure S5**). When alpha diversity was assessed using the Chao1 index, neither soil water content (*F*_1,10_ = 1.841, *p* = 0.2046), nor any of the measures of soil texture (% silt: *F*_1,10_ = 0.2584, *p* = 0.6222, % clay: *F*_1,10_ = 2.827, *p* = 0.1236, and % sand: *F*_1,10_ = 0.5294, *p* = 0.4835) significantly altered alpha diversity (**Supplementary Figures S6B,E,H,K**). When alpha diversity was assessed using the Shannon index, % silt significantly decreased alpha diversity (*F*_1,10_ = 6.533, *p* = 0.0286), but soil water content (*F*_1,10_ = 0.4496, *p* = 0.5177), % sand (*F*_1,10_ = 1.794, *p* = 0.2101), and % clay (*F*_1,10_ = 1.466, *p* = 0.2538) did not significantly alter alpha diversity (**Supplementary Figures S5C,F,I,L**). When % silt and % sand were combined into a single metric, results echoed these patterns (**Supplementary Figure S6**). When % sand and % silt were combined into a single metric, the ratio significantly increased alpha diversity as assessed by the Shannon diversity index, in line with the fact that % silt alone decreased the Shannon index.

**FIGURE 3:**
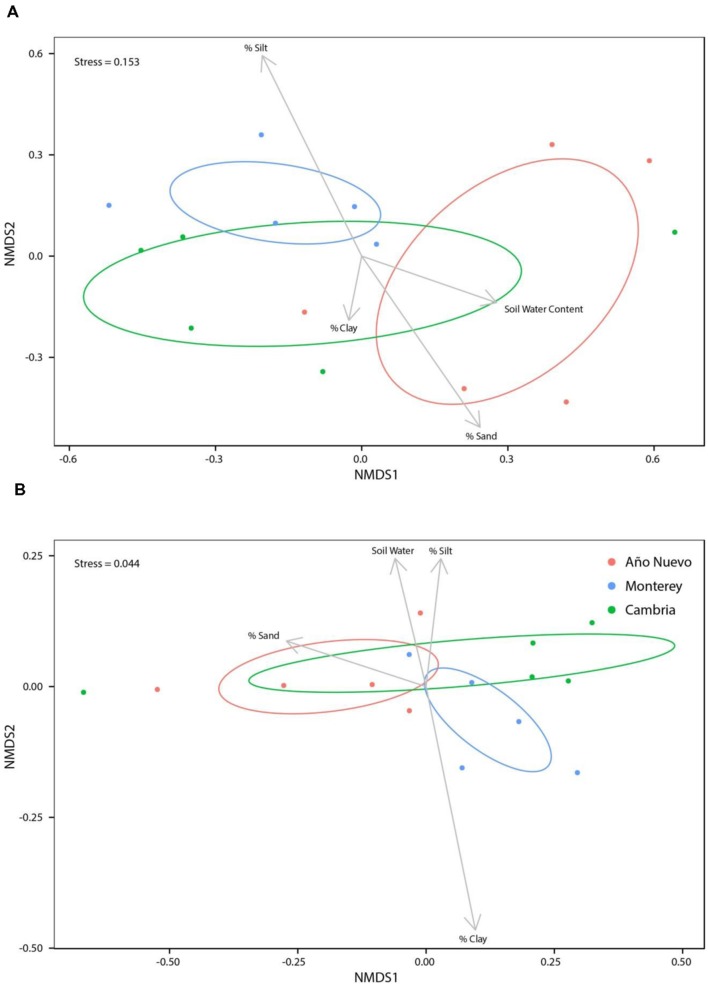
**Non-metric multidimensional scaling ordinations for bacteria **(A)** and fungi **(B)** communities as a function of environmental variables.** Ellipses indicate 95% confidence intervals for samples collected from Año Nuevo (salmon), Monterey (green), and Cambria (blue). Arrows specify association of environmental variables with particular sites.

While soil water content (*R*^2^ = 0.14, *p* = 0.096), % sand (*R*^2^ = 0.07, *p* = 0.310), and % clay (*R*^2^ = 0.03, *p* = 0.694) were not significant structuring factors of bacterial assemblages, % silt was important for structuring bacterial assemblages (*R*^2^ = 0.17, *p* = 0.049, **Figure 3A**). When % silt and % sand were combined into a single metric, none of the environmental characteristics were significant structuring factors of bacterial assemblages [**Supplementary Figure S7**; soil water content (*R*^2^ = 0.14, *p* = 0.08), % sand: % silt (*R*^2^ = 0.15, *p* = 0.062), and % clay (*R*^2^ = 0.13, *p* = 0.087)]. No bacterial OTUs were correlated (>0.5) with soil water content (**Supplementary Figure S8**). There were no strong correlations between bacterial OTUs and soil texture but some OTUs tended to prefer specific soil characteristics. While no OTUs were negatively correlated with % clay, Acetobacteraceae6 (0.6131) tended to be positively correlated with % clay (**Supplementary Figure S8**). *Sphingomonas2* (0.7343) and Methylocystaceae2 (0.6021) tended to be positively correlated with % silt and Enterobacteriaceae9 (-0.6962) tended to be negatively correlated with % silt. No OTUs were correlated with % sand (**Supplementary Figure S8**).

When % silt and % sand were combined into a single metric, correlations of OTUs with environmental characteristics changed marginally. One bacterial OTU was moderately positively correlated (>0.5) with soil water content (Burkholderiales2: 0.5338, **Supplementary Figure S10**). Echoing results when environmental variables were analyzed separately, there were no strong correlations between bacterial OTUs and soil texture but some OTUs tended to prefer specific soil characteristics. *Sphingomonas2* (-0.5474) was negatively correlated with % clay, and Cystobacterineae2 (0.5261), Enterobacteriaceae9 (0.5527), and Acetobacteraceae6 (0.6131) tended to be positively correlated with % clay (**Supplementary Figure S9**). *Sphingomonas2* (-0.5152) tended to be negatively correlated with % sand: % silt and *Janthinobacterium* (0.5107), Acetobacteraceae16 (0.547), and Enterobacteriaceae9 (0.5918) tended to be positively correlated with % sand: % silt (**Supplementary Figure S9**).

#### Community Structure as a Function of Tissue Type

Alpha diversity differed between tissue type (bud vs. needle) when assessed using the Chao1 index (*F*_1,4_ = 13.09, *p* = 0.0224) but not when assessed using the Shannon index (*F*_1,4_ = 0.5922, *p* = 0.4845, **Supplementary Figure S10**).

Tissue type was a weak structuring factor of bacterial assemblages (*R*^2^ = 0.44, *p* = 0.10) and communities tended to group accordingly, although there was a fair amount of scatter in NMDS ordinations (**Supplementary Figure S11**). No bacterial species were significantly associated with either tissue type despite Bdellovibrionaceae, Methylobacteriaceae, and Salinisphaeraceae being restricted to buds only.

### ECM Fungal Species

#### Overall Community Description

The ECM fungal community was identified from a total of 6792 colonized root tips (Año Nuevo: 1976, Monterey: 2478, Cambria: 2338), and consisted of 88 OTUs from 32 families (**Figure 1B**). The fungal sequence data for this project have been submitted to the GenBank databases under the accession numbers KU509057 – KU509213. The three most common families were Cortinariaceae (23% of total tips), Thelephoraceae (22% of total tips), and Russulaceae (16% of total tips).

#### Community Structure as a Function of Population

No difference in alpha diversity was found among locations for either the Chao1 index (*F*_2,12_ = 0.1368, *p* = 0.8734) or the Shannon index (*F*_2,12_ = 0.1332, *p* = 0.8766, **Supplementary Figure S4D–F**).

Mycorrhizal community abundances were sorted into an ordination plot according to community similarity (**Figure 2B**). Population was a significant structuring factor of mycorrhizal assemblages (*R*^2^ = 0.19, *p* = 0.007), and communities tended to group accordingly (**Figure 2B**). *Cortinarius2* was strongly and significantly associated with Monterey (*p* = 0.012), while no OTUs were significantly associated with either Cambria or Año Nuevo.

#### Community Structure as a Function of Environmental Characteristics

No difference in alpha diversity was found among environmental characteristics for either the Chao1 index (soil water content: *F*_1,10_ = 0.0036, *p* = 0.9532; % silt: *F*_1,10_ = 0.0977, *p* = 0.7610; % sand: *F*_1,10_ = 2.102, *p* = 0.1778; % clay: *F*_1,10_ = 0.0998, *p* = 0.7586) or the Shannon index (soil water content: *F*_1,10_ = 0.2526, *p* = 0.6261; % silt: *F*_1,10_ = 0.0981, *p* = 0.7606; % sand: *F*_1,10_ = 0.7501, *p* = 0.4068; % clay: *F*_1,10_ = 0.0258, *p* = 0.8756, **Supplementary Figure S12**). When % silt and % sand were combined into a single metric, results echoed these patterns (**Supplementary Figure S13**).

Soil water content was a significant structuring factor of mycorrhizal assemblages (*R*^2^ = 0.09, *p* = 0.039) but none of the measures of soil texture (% silt: *R*^2^ = 0.06, *p* = 0.843, % sand: *R*^2^ = 0.08, *p* = 0.295, % clay: *R*^2^ = 0.07, *p* = 0.550) were significant structuring factors (**Figure 3B**). When % silt and % sand were combined into a single metric, results echoed these patterns (**Supplementary Figure S14**). Correlation analysis indicated that three fungal OTUs were highly correlated with soil water content [*Tuber gibbosum* (0.7335), *Paratritirachium* (0.7335), *Russula8* (0.7335)] but no OTUs were negatively correlated with soil water content (**Supplementary Figure S10**). No fungal OTUs were significantly correlated with % clay or % sand, but *Cortinarius2* (0.6053) was positively correlated with % silt (**Supplementary Figure S15**). When % silt and % sand were combined into a single metric, correlations of OTUs with soil water content did not change (**Supplementary Figure S16**), but correlations with environmental characteristics changed marginally such that *Russula4* (0.5439), *Russula5* (0.5439), *Russula6* (0.5439) and Inocybaceae (0.5439) were positively correlated with % sand: % silt (**Supplementary Figure S16**). Echoing results in which environmental variables are analyzed separately, no fungal OTUs were significantly correlated with % clay (**Supplementary Figure S16**).

#### Distribution of Fungal Traits Involved with Foraging Strategy

The distribution of fungal traits associated with foraging strategy was dependent upon population for exploration type (χ^2^ = 254.2, *p* < 0.0001), rhizomorph production (χ^2^ = 30.88, *p* < 0.0001), and hydrophobicity (χ^2^ = 30.88, *p* < 0.0001). Specifically, ECM fungi with the medium distance exploration type were most prominent at Monterey and Cambria but ECM fungi with the short distance exploration type were more prominent at Año Nuevo (**Supplementary Figure S17**). Additionally, ECM fungi at Monterey were more likely to produce rhizomorphs and be hydrophobic while the reverse was true at Año Nuevo where ECM were less likely to produce rhizomorphs and be hydrophilic. There was no difference in either fungal trait for fungi at Cambria.

Exploration type of the ECM fungi significantly interacted with several of the environmental factors to influence fungal frequency (Soil Water Content: *p* < 0.0001, % sand: *p* < 0.0001, % silt: *p* < 0.0001, % clay: *p* < 0.0001). Rhizomorph production significantly interacted with several of the environmental factors to influence fungal frequency (Soil Water Content: *p* = 0.0.0054, % sand: *p* < 0.0001, % silt: *p* < 0.0001, % clay: *p* = 0.1241). Hydrophobicity significantly interacted with several of the environmental factors to influence fungal frequency (Soil Water Content: *p* = 0.0054, % sand: *p* < 0.0001, % silt *p* < 0.0001, % clay: *p* = 0.1241).

### Interaction of Bacterial Endophytes and ECM Fungi

**FIGURE 4:**
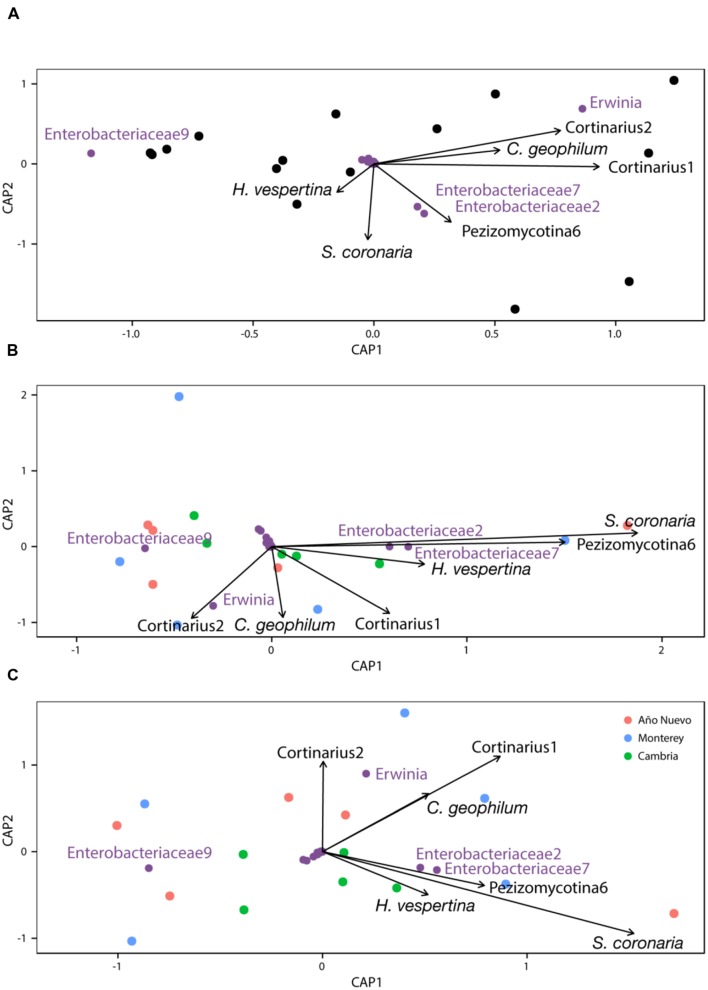
**Constrained analysis of principal coordinates with no conditions **(A)**, conditioned by population **(B)**, and conditioned by environment **(C)****.

CAP analysis with Type I ANOVA indicates that *Cortinarius1* (*F*_1,8_ = 4.678, *p* = 0.013) was an important structuring factor for bacterial assemblages and *Sarcosphaera coronaria* (*F*_1,8_ = 2.399, *p* = 0.074) tended to be important as well (**Figure 4A**). This relationship changed slightly when the model was conditioned for population before constraining for other variables (*Cortinarius1*: *F*_1,8_ = 2.9, *p* = 0.069, *Sarcosphaera coronaria: F*_1,8_ = 3.314, *p* = 0.048, Pezizomycotina 6: *F*_1,8_ = 2.973, *p* = 0.087, **Figure 4B**) and when the model was conditioned for the environmental characteristics before constraining for other variables (*Cortinarius1*: *F*_1,8_ = 2.889, *p* = 0.075, *Sarcosphaera coronaria: F*_1,8_ = 2.8, *p* = 0.058, **Figure 4C**).

We also investigated the role foraging traits play in structuring bacterial endophyte communities. None of the possible exploration types significantly structured bacterial assemblages (**Table 2**); however, the long distance exploration type tended to be associated with the bacterial species Enterobacteriaceae9 and the medium distance type tended to be associated with Erwinia (**Supplementary Figure S18**). Neither rhizomorph production nor hydrophobicity significantly structured bacterial assemblages (**Table 2**). Conditioning the model with population or the environmental characteristics before constraining for other variables changed these results such that the long distance exploration type tended to be important for structuring the bacterial assemblages (*F*_1,6_ = 2.294, *p* = 0.092, **Table 2**).

**Table 2:** ANOVA tables from CAP models describing the relationship between fungal traits describing foraging strategy and bacterial assemblages.

	Default				Population				Environmental Characteristics			
			
	Df	SS	*F*	Pr(>F)	Df	SS	*F*	Pr(>F)	Df	SS	*F*	Pr(>F)
**Exploration type**							
Contact	1	0.0989	0.4655	0.782	1	0.1435	0.9495	0.425	1	0.2191	1.551	0.217
Long distance	1	0.1516	0.7137	0.559	1	0.1303	0.8620	0.435	1	0.3241	2.294	0.092
Medium distance	1	0.1352	0.6363	0.602	1	0.0372	0.2460	0.937	1	0.0362	0.2561	0.889
Short distance	1	0.0767	0.3612	0.843	1	0.0722	0.4780	0.751	1	0.1300	0.9201	0.448
Residual	10	2.1247			8	1.2090			6	0.8476		
**Rhizomorph production**							
No Rhizomorphs	1	0.0353	0.1838	0.966	1	0.0373	0.2601	0.931	1	0.0522	0.3241	0.856
Rhizo-forming	1	0.2456	1.278	0.255	1	0.1206	0.8407	0.488	1	0.2153	1.336	0.266
Residual	12	2.3063			10	1.4343			8	1.2894		
**Hydrophobicity**							
Hydrophilic	1	0.0353	0.1838	0.969	1	0.0373	0.2601	0.923	1	0.0522	0.3241	0.876
Hydrophobic	1	0.2456	1.2779	0.303	1	0.1206	0.8407	0.478	1	0.2153	1.336	0.268
Residual	12	2.3063			10	1.4343			8	1.2894		


*The p-value represents the probability of a value of F greater than or equal to the observed value. No foraging strategies were significantly associated (at *p* = 0.05) with describing variation in bacterial assemblages for the default model or models conditioned with either population or environmental characteristics. Italicized values represent significance between *p* = 0.05 and *p* = 0.1. Df, degrees of freedom; SS, sums of squares; F, F-statistic.*

## Discussion

The plant microbiome is highly diverse, yet little is understood regarding the interaction of microbes from multiple guilds. Our results suggest the potential for relationships between specific ECM fungi and bacterial endophyte communities that are likely to be influenced by population and environmental characteristics. Specifically, we found that the fungal OTU *Cortinarius1* was significant in structuring the bacterial endophyte community, and that the OTU *Sarcosphaera coronaria* was marginally significant, but that when either population or environmental characteristics were taken into account, the importance of particular ECM fungi for structuring bacterial communities changed such that *Cortinarius1* became a less important factor and *S. coronaria* became a more important factor. These results justify further research into the relationship between fungal and bacterial components of the plant microbiome.

The ecological and evolutionary implications of these results are important as the processes and patterns which structure microbial communities remains an area of developing research. The advancement of molecular methodology has extended our knowledge of the factors that influence aspects of microbial communities including diversity, niche partitioning, competition, spatial variability, and functional traits (Peay et al., 2008; Poole et al., 2012), but the role microbial species interactions play in altering these factors is an area of developing research. For example, ecological theory indicates that species interactions may either enlarge the realized niches of species (facilitation; Bruno et al., 2003) or contract the realized niche (competition; Pianka, 1981) but these concepts have yet to be extended to include the interactions of microbes from multiple guilds (e.g., fungi and bacteria). Furthermore, the role of environmental variables in influencing multispecies interactions is generally neglected. Our research indicates that, in addition to abiotic variables, consideration of biotic interactions, such as those between bacterial endophytes and ECM fungi, may be important for understanding the factors that structure microbial communities.

### Foraging Traits of ECM Fungi as Structuring Forces

Fungal life-history traits such as those associated with foraging strategy are likely to provide important insight into how species interactions may ultimately shape microbial communities. Across populations and environmental factors, none of the traits associated with fungal foraging strategy types were significantly associated with bacterial assemblages as a whole but two bacterial species were more likely to be associated with specific ECM fungal traits associated with foraging strategy. Members of the bacterial genus *Erwinia* were correlated to the abundance of ECM from the medium exploration type while members of the family Enterobacteriaceae9 were correlated to long distance exploration types. ECM fungi belonging to the long distance exploration type make a higher investment in transport mycelium, indicating a higher carbon cost to their host than taxa that form less carbon intensive rhizomorphs such as those from the short, contact and some medium distance exploration types. Therefore, conditions that favor greater belowground carbon allocation are likely to favor long distance exploration types while conditions which favor low belowground carbon allocation are likely to favor other exploration types. The dominant bacteria in this study – Enterobacteriaceae and *Erwinia* – could in theory be mutualists, commensal, or opportunistic pathogens of Monterey pine. The presence of bacteria that alleviate plant stress or promote plant growth could allow for conditions that favor greater belowground carbon allocation, leading to an increased presence of long distance exploration fungal types. On the other hand, these bacteria could be opportunists which take advantage of the elevated drought stress Monterey pine populations are currently experiencing (Hoeksema and Rúa, unpublished data). While using a trait-based approach to ECM fungal ecology allows us to link ECM diversity to important ecosystem processes such as nitrogen and carbon cycling (Krause et al., 2014; Aguilar-Trigueros et al., 2015), in order to fully understand the ecological and evolutionary implications of such interactions, further work utilizing eco-physiological studies to examine the potential mechanisms behind these interactions is needed.

Interestingly, it is only after accounting for variation due to environmental factors that the long distance exploration type tends to be associated with bacterial communities. This is likely because each of the environmental characteristics we measured interacted with the foraging traits to significantly alter the frequency of ECM fungi. Previous studies have found that environmental characteristics can shape the presence and proportion of different ECM exploration types (Suz et al., 2014). In our system, the abundance of the contact exploration type was highest at Monterey and Cambria, suggesting conditions at those sites favoring fungi able to access nutrients from labile nutrient pools, while Año Nuevo was dominated by fungi classified as the long exploration type, suggesting conditions at those sites favoring fungi able to access nutrients from recalcitrant nutrient pools (**Supplementary Figure S17**) (Lilleskov et al., 2002; Hobbie and Agerer, 2010). Interestingly, the environmental characteristics we measured were more closely related for Año Nuevo and Cambria than Monterey and Cambria (**Supplementary Figures S1–S3**), suggesting nutrient availability may not track with our environmental characteristics. These results suggest it is not only species interactions but also abiotic factors that may be important for understanding microbial community dynamics in forest ecosystems.

### Bacterial Endophyte Community Composition

While the needle endophyte communities of other conifers surveyed to date have been found to consist largely of bacteria in the lineages Alphaproteobacteria, Acidobacteria, and Firmicutes (Carrell and Frank, 2014, 2015), the endophyte communities of Monterey pine needles in this study consisted largely of Gammaproteobacteria. Across all plant species studied, this class of bacteria is the one most commonly found in endophyte communities (Hardoim et al., 2015). The majority of samples across all three populations were dominated by one or several of four OTUs in the *Enterobacteriaceae*, a family that is commonly associated with plants, both as mutualists and pathogens. Due to horizontal gene transfer, bacterial groups encompass large diversity in genome content, making it impossible to infer physiology from taxonomic identity at the family or even species level. Mutualistic species in this family, including *Klebsiella*, *Enterobacter*, and *Rahnella* spp., have been found to promote growth, solubilize phosphate, fix nitrogen, and protect plants against fungal pathogens and drought (Chernin et al., 1995; Fouts et al., 2008; Hardoim et al., 2013; Naveed et al., 2014; Upreti and Thomas, 2015). Pathogenic Enterobacteriaceae include *Erwinia amylovora*, the causative agent of fire blight on apple trees, *Erwinia pyrifoliae*, which causes bacterial shoot blight in pear, and *Erwinia carotovora*, the causative agent of soft rot and blackleg potato diseases (Rhim et al., 1999; Shrestha et al., 2003; Bell et al., 2004); however, *Erwinia* spp. have also been found as endophytes in apparently healthy plants, including in high relative abundance in healthy maples growing in an urban environment (Shen and Fulthorpe, 2015). At this stage, we cannot determine whether the dominant *Enterobacteriacae* in the Monterey pine foliar endophyte community interacts with the hosts as mutualists, commensals or opportunistic pathogens. The dominance of these taxa as bacterial endophytes could be the result of beneficial interactions, for example through buffering of abiotic stresses imposed on the plant by their harsh coastal environments. Alternatively, they could represent opportunistic bacteria taking advantage of drought-stressed trees. The extent to which selection, dispersal, and drift shape these communities is largely unknown, but it is possible that ecological drift events could influence above-ground endophyte communities over relatively short time scales. For example, immigrated dust-associated bacteria can drastically alter the communities of bacterial communities in the phyllosphere (Rastogi et al., 2012), and significant variation in foliar endophyte communities across seasons has been observed (Shen and Fulthorpe, 2015).

With only 83 OTUs identified, the foliar endophyte community of Monterey pine was less diverse than that of other conifers surveyed with next-generation 16S rRNA sequencing (Carrell and Frank, 2014, 2015), but rarefaction analysis indicated that we did not under-sample the community (**Supplementary Figure S3**). Interestingly, the Gammaproteobacteria from Monterey pine were more readily cultured than bacteria from high elevation pines (Wilson and Frank, unpublished), potentially consistent with opportunistic invasion of Gammaproteobacteria. Further insight into the structure and role of Monterey pine foliar endophyte communities could be resolved with surveys across multiple time points as well as controlled inoculation experiments.

The buds of *P. sylvestris* have been found to consistently host growth-promoting, intracellular bacteria with a potential role in bud elongation and differentiation (Pirttilä et al., 2000, 2004, 2005; Pohjanen et al., 2014). Although bud and needle communities of *P. radiata* in this study were not significantly different as a whole, we identified three bacterial families unique to buds: Bdellovibrionaceae, Methylobacteriaceae, and Salinisphaeraceae. Members of the Bdellovibrionaceae are unique among bacteria in that they often parasitize other Gram-negative bacteria, and have been identified previously as endophytes in hybrid maize (Liu et al., 2012) while members of the Salinisphaeraceae family exhibit extremely high levels of salt tolerance (Vetriani et al., 2014). To our knowledge, members of the Salinisphaeraceae have not previously been identified as endophytes. Given that all members of this family have been isolated from marine and high-salinity environments (Vetriani et al., 2014), the presence of this taxon in Monterey pine buds likely reflects the coastal habitat. Finally, identification of members of the Methylobacteriaceae complements previous research investigating bacterial endophytes in buds, which found that *M. extorquens* DSM 1360 is a dominant species in *P. sylvestris* buds throughout the year (Pirttilä et al., 2000, 2005).

Environmental factors were important for shaping bacterial endophyte communities not only by mediating their interaction with ECM fungi but also by directly altering their distribution, emphasizing the important role environmental filtering plays in shaping these communities. Specifically, % clay and soil water content both decreased bacterial alpha diversity, stressing the potential importance of water availability for shaping these communities. Representative species of the family *Enterobacteriace*, including *Klebsiella, Enterobacter*, and *Citrobacter*, have all been isolated from plant species grown in arid environments with similar environmental characteristics (Hayat et al., 2010; Marasco et al., 2012), indicating the potential for endophytes from this class to be advantageous under adverse environmental conditions. Interestingly, in our study system, % silt and % sand were found to be correlated (**Table 1**) but when combined into a single metric, results were not qualitatively different then when left apart (see Supplementary Material). The main effect of combining % silt and % sand into a single metric was to marginally alter the magnitude of correlations of bacterial and fungal OTUs with respect to soil texture (**Supplementary Figures S9** and **S16**).

### ECM Fungal Community

Contrary to previous work examining ECM communities in *P. radiata* (Hoeksema et al., 2012), we did not find significant differences in species richness among the populations (**Supplementary Figure S4D–F**) but population was important for understanding differences in community structure. The ECM fungal community in Monterey was different from those found at Año Nuevo or Cambria (**Supplementary Figure S4D–F**), which is likely due to differences in the sites’ environmental characteristics. Monterey had the lowest soil water content among the three sites and differed greatly from the other sites in all three soil texture measurements (lowest % clay, lowest % sand, and highest % silt, **Supplementary Figure S1**). Interestingly, the OTU *Cortinarius2* was both a significant indicator of the Monterey population and highly positively correlated with the % silt in the soil but not significantly related to % sand as might be expected since these environmental variables are correlated in this system (**Table 1**); however, previous work with the *Cortinarius* genus indicates members of this genus play an important role in the decomposition of complex organic matter (Hobbie et al., 2013; Bödeker et al., 2014), suggesting this functional trait may be important in high silt environments like Monterey.

## Conclusion

Understanding the ecological and evolutionary dynamics that shape plant microbial communities is important for both natural and agroecosystems as well as a wide range of fields, including biotechnology and sustainable agriculture; however, in order to more fully understand these processes, research which explores the variation in microbial community structure due to interactions among microbes is vital (Dickie et al., 2012; Hanson et al., 2012; Rillig et al., 2015). By uniting two ubiquitous microbial communities, this research represents a first crucial step toward understanding such interactions in forests.

## Author Contributions

MR, EW, and AF conceived and designed the experiment. MR and EW collected field samples; MR and SS performed fungal laboratory analyses; EW and AM performed bacterial laboratory analyses; MR analyzed the data with input from AF; MR, and AF contributed reagents/materials/analysis tools; MR wrote the paper. All authors revised the manuscript critically for important intellectual content and approved the final version of the manuscript.

## Conflict of Interest Statement

The authors declare that the research was conducted in the absence of any commercial or financial relationships that could be construed as a potential conflict of interest.

## References

[B1] Agerer R. (2001). Exploration types of ectomycorrhizae. *Mycorrhiza* 11 107–114. 10.1007/s005720100108

[B2] Agerer R. (2006). Fungal relationships and structural identity of their ectomycorrhizae. *Mycol. Progr.* 5 67–107. 10.1007/s11557-006-0505-x

[B3] Aguilar-Trigueros C. A. Hempel S. Powell J. R. Anderson I. C. Antonovics J. Bergmann J. (2015). Branching out: towards a trait-based understanding of fungal ecology. *Fungal Biol. Rev.* 29 34–41. 10.1016/j.fbr.2015.03.001

[B4] Altschul S. F. Gish W. Miller W. Myers E. W. Lipman D. J. (1990). Basic local alignment search tool. *J. Mol. Biol.* 215 403–410. 10.1016/S0022-2836(05)80360-2 2231712

[B5] Anderson I. C. Cairney J. W. G. (2007). Ectomycorrhizal fungi: exploring the mycelial frontier. *FEMS Microbiol. Rev.* 31 388–406. 10.1111/j.1574-6976.2007.00073.x 17466031

[B6] Arnold A. E. Henk D. A. Eells R. L. Lutzoni F. Vilgalys R. (2007). Diversity and Phylogenetic affinities of foliar fungal endophytes in loblolly pine inferred by culturing and environmental PCR. *Mycologia* 99 185–206. 10.3852/mycologia.99.2.185 17682771

[B7] Aronesty E. (2011). *ea-utils : Command-line Tools for Processing Biological Sequencing Data*. Available at: http://code.google.com/p/ea-utils

[B8] Axelrod D. I. (1986). Cenozoic history of some western american pines. *Ann. Missouri Bot. Garden* 73 565–641. 10.2307/2399194

[B9] Bell K. S. Sebaihia M. Pritchard L. Holden M. T. G. Hyman L. J. Holeva M. C. (2004). Genome sequence of the enterobacterial phytopathogen Erwinia carotovora subsp. atroseptica and characterization of virulence factors. *Proc. Natl. Acad. Sci. U.S.A.* 101 11105–11110. 10.1073/pnas.0402424101 15263089 PMC503747

[B10] Bever J. D. (2003). Soil community feedback and the coexistence of competitors: conceptual frameworks and empirical tests. *New Phytol.* 157 465–473. 10.1046/j.1469-8137.2003.00714.x 33873396

[B11] Bödeker I. T. M. Clemmensen K. E. de Boer W. Martin F. Olson Å. Lindahl B. D. (2014). Ectomycorrhizal Cortinarius species participate in enzymatic oxidation of humus in northern forest ecosystems. *New Phytol.* 203 245–256. 10.1111/nph.12791 24725281

[B12] Bokulich N. A. Subramanian S. Faith J. J. Gevers D. Gordon J. I. Knight R. (2013). Quality-filtering vastly improves diversity estimates from Illumina amplicon sequencing. *Nat. Methods* 10 57–59. 10.1038/nmeth.2276 23202435 PMC3531572

[B13] Bruno J. F. Stachowicz J. J. Bertness M. D. (2003). Inclusion of facilitation into ecological theory. *Trends Ecol. Evol.* 18 119–125. 10.1016/S0169-5347(02)00045-9

[B14] Caporaso J. G. Bittinger K. Bushman F. D. DeSantis T. Z. Andersen G. L. Knight R. (2010a). PyNAST: a flexible tool for aligning sequences to a template alignment. *Bioinformatics* 26 266–267. 10.1093/bioinformatics/btp636 19914921 PMC2804299

[B15] Caporaso J. G. Kuczynski J. Stombaugh J. Bittinger K. Bushman F. D. Costello E. K. (2010b). QIIME allows analysis of high-throughput community sequencing data. *Nat. Methods* 7 335–336. 10.1038/nmeth.f.303 20383131 PMC3156573

[B16] Carrell A. A. Frank A. C. (2014). *Pinus flexilis* and *Piceae engelmannii* share a simple and consistent needle endophyte microbiota with a potential role in nitrogen fixation. *Front. Microbiol.* 5:333 10.3389/fmicb.2014.00333. PMC4082182 25071746

[B17] Carrell A. A. Frank A. C. (2015). Bacterial endophyte communities in the foliage of coast redwood and giant sequoia. *Front. Microbiol.* 6:e01008 10.3389/fmicb.2015.01008 PMC4585279 26441933

[B18] Carroll G. (1988). Fungal endophytes in stems and leaves: from latent pathogen to mutualistic symbiont. *Ecology* 69 2–9. 10.2307/1943154

[B19] Chanway C. P. (1996). Endophytes: they’re not just fungi! *Can. J. Bot.* 74 321–322. 10.1139/b96-040

[B20] Chanway C. P. Anand R. Yang H. (2014). “Nitrogen fixation outside and inside plant tissues,” in *Advances in Biology and Ecology of Nitrogen Fixation*, ed. Ohyama T. (InTech).

[B21] Chelius M. K. Triplett E. W. (2001). The diversity of archaea and bacteria in association with the roots of Zea mays L. *Microb. Ecol.* 41 252–263. 10.1007/s002480000087 11391463

[B22] Chernin L. Ismailov Z. Haran S. Chet I. (1995). Chitinolytic enterobacter agglomerans antagonistic to fungal plant pathogens. *Appl. Environ. Microbiol.* 61 1720–1726. 16535017 10.1128/aem.61.5.1720-1726.1995 PMC1388435

[B23] De Cáceres M. Legendre P. (2009). Associations between species and groups of sites: indices and statistical inference. *Ecology* 90 3566–3574. 10.1890/08-1823.1 20120823

[B24] De Cáceres M. Legendre P. Moretti M. (2010). Improving indicator species analysis by combining groups of sites. *Oikos* 119 1674–1684. 10.1111/j.1600-0706.2010.18334.x

[B25] de Mendiburu F. (2014). *Agricolae: Statistical Procedures for Agricultural Research*. R package version 1.2-1 Available at: http://CRAN.R-project.org/package=agricolae

[B26] DeSantis T. Z. Hugenholtz P. Larsen N. Rojas M. Brodie E. L. Keller K. (2006). Greengenes, a chimera-checked 16S rRNA gene database and workbench compatible with ARB. *Appl. Environ. Microbiol.* 72 5069–5072. 10.1128/aem.03006-3005 16820507 PMC1489311

[B27] Dickie I. A. Fukami T. Wilkie J. P. Allen R. B. Buchanan P. K. (2012). Do assembly history effects attenuate from species to ecosystem properties? A field test with wood-inhabiting fungi. *Ecol. Lett.* 15 133–141. 10.1111/j.1461-0248.2011.01722.x 22188588

[B28] Edgar R. C. (2010). Search and clustering orders of magnitude faster than BLAST. *Bioinformatics* 26 2460–2461. 10.1093/bioinformatics/btq461 20709691

[B29] Edgar R. C. (2013). UPARSE: highly accurate OTU sequences from microbial amplicon reads. *Nat. Methods* 10 996–998. 10.1038/nmeth.2604 23955772

[B30] Fogel R. (1988). Interactions among soil biota in coniferous ecosystems. *Agricult. Ecosyst. Environ.* 24 69–85. 10.1016/0167-8809(88)90057-6

[B31] Fouts D. E. Tyler H. L. DeBoy R. T. Daugherty S. Ren Q. Badger J. H. (2008). Complete genome sequence of the N_2_-fixing broad host range endophyte *Klebsiella pneumoniae* 342 and virulence predictions verified in mice. *PLoS Genet.* 4:e1000141 10.1371/journal.pgen.1000141 PMC2453333 18654632

[B32] Gardes M. Bruns T. D. (1993). ITS primers with enhanced specificity for basidiomycetes—application to the identification of mycorrhizae and rusts. *Mol. Ecol.* 2 113–118. 10.1111/j.1365-294X.1993.tb00005.x 8180733

[B33] Griffin E. Carson W. (2015). The ecology and natural history of foliar bacteria with a focus on tropical forests and agroecosystems. *Bot. Rev.* 81 105–149. 10.1007/s12229-015-9151-9159

[B34] Grotkopp E. Rejmánek M. Sanderson M. J. Rost T. L. (2004). Evolution of genome size in Pines (Pinus) and its life-history correlates: supertree analyses. *Evolution* 58 1705–1729. 10.1111/j.0014-3820.2004.tb00456.x 15446425

[B35] Hanson C. A. Fuhrman J. A. Horner-Devine M. C. Martiny J. B. H. (2012). Beyond biogeographic patterns: processes shaping the microbial landscape. *Nat. Rev. Microbiol.* 10 497–506. 10.1038/nrmicro2795 22580365

[B36] Hardoim P. Nazir R. Sessitsch A. Elhottová D. Korenblum E. van Overbeek L. (2013). The new species *Enterobacter oryziphilus* sp. nov. and *Enterobacter oryzendophyticus* sp. nov. are key inhabitants of the endosphere of rice. *BMC Microbiol.* 13:164 10.1186/1471-2180-13-164 PMC3728145 23865888

[B37] Hardoim P. R. van Overbeek L. S. Berg G. Pirttilä A. M. Compant S. Campisano A. (2015). The hidden world within plants: ecological and evolutionary considerations for defining functioning of microbial endophytes. *Microbiol. Mol. Biol. Rev.* 79 293–320. 10.1128/mmbr.00050-14 26136581 PMC4488371

[B38] Hayat R. Ali S. Amara U. Khalid R. Ahmed I. (2010). Soil beneficial bacteria and their role in plant growth promotion: a review. *Ann. Microbiol.* 60 579–598. 10.1007/s13213-010-0117-111

[B39] Hobbie E. A. Agerer R. (2010). Nitrogen isotopes in ectomycorrhizal sporocarps correspond to belowground exploration types. *Plant Soil* 327 71–83. 10.1007/s11104-009-0032-z

[B40] Hobbie E. A. Ouimette A. P. Schuur E. A. G. Kierstead D. Trappe J. M. Bendiksen K. (2013). Radiocarbon evidence for the mining of organic nitrogen from soil by mycorrhizal fungi. *Biogeochemistry* 114 381–389. 10.1007/s10533-012-9779-z

[B41] Hoeksema J. D. Hernandez J. V. Rogers D. L. Mendoza L. L. Thompson J. N. (2012). Geographic divergence in a species-rich symbiosis: interactions between Monterey pines and ectomycorrhizal fungi. *Ecology* 93 2274–2285. 10.1890/11-1715.1 23185888

[B42] Hoeksema J. D. Thompson J. N. (2007). Geographic structure in a widespread plant–mycorrhizal interaction: pines and false truffles. *J. Evol. Biol.* 20 1148–1163. 10.1111/j.1420-9101.2006.01287.x 17465924

[B43] Horton T. R. (2002). Molecular approaches to ectomycorrhizal diversity studies: variation in ITS at a local scale. *Plant Soil* 244 29–39. 10.1023/A:1020268020563

[B44] Huang X. Madan A. (1999). Cap3: a DNA sequence assembly program. *Genome Res.* 9 868–877. 10.1101/gr.9.9.868 10508846 PMC310812

[B45] Innerebner G. Knief C. Vorholt J. A. (2011). Protection of *Arabidopsis thaliana* against Leaf-Pathogenic *Pseudomonas syringae* by sphingomonas strains in a controlled model system. *Appl. Environ. Microbiol.* 77 3202–3210. 10.1128/aem.00133-111 21421777 PMC3126462

[B46] Izzo A. Agbowo J. Bruns T. D. (2005). Detection of plot-level changes in ectomycorrhizal communities across years in an old-growth mixed-conifer forest. *New Phytol.* 166 619–630. 10.1111/j.1469-8137.2005.01354.x 15819924

[B47] James E. K. (2000). Nitrogen fixation in endophytic and associative symbiosis. *Field Crops Res.* 65 197–209. 10.1016/S0378-4290(99)00087-8

[B48] Ji P. Wilson M. (2002). Assessment of the importance of similarity in carbon source utilization profiles between the biological control agent and the pathogen in biological control of bacterial speck of tomato. *Appl. Environ. Microbiol.* 68 4383–4389. 10.1128/aem.68.9.4383-4389.2002 12200291 PMC124063

[B49] Jiao J. Y. Wang H. X. Zeng Y. Shen Y. M. (2006). Enrichment for microbes living in association with plant tissues. *J. Appl. Microbiol.* 100 830–837. 10.1111/j.1365-2672.2006.02830.x 16553739

[B50] Kõljalg U. Nilsson R. H. Abarenkov K. Tedersoo L. Taylor A. F. S. Bahram M. (2013). Towards a unified paradigm for sequence-based identification of fungi. *Mol. Ecol.* 22 5271–5277. 10.1111/mec.12481 24112409

[B51] Koskimäki J. J. Pirttilä A. M. Ihantola E.-L. Halonen O. Frank A. C. (2015). The intracellular scots pine shoot symbiont methylobacterium extorquens dsm13060 aggregates around the host nucleus and encodes eukaryote-like proteins. *mBio* 6:e00039 10.1128/mBio.00039-15. PMC4453540 25805725

[B52] Krause S. Le Roux X. Niklaus P. A. Van Bodegom P. M. Lennon J. T. Bertilsson S. (2014). Trait-based approaches for understanding microbial biodiversity and ecosystem functioning. *Front. Microbiol.* 5:251 10.3389/fmicb.2014.00251 PMC4033906 24904563

[B53] Lepage B. Currah R. Stockey R. Rothwell G. (1997). Fossil ectomycorrhizae from the Middle Eocene. *Am. J. Bot.* 84:410 10.2307/2446014 21708594

[B54] Lilleskov E. A. Hobbie E. A. Fahey T. J. (2002). Ectomycorrhizal fungal taxa differing in response to nitrogen deposition also differ in pure culture organic nitrogen use and natural abundance of nitrogen isotopes. *New Phytol.* 154 219–231. 10.1046/j.1469-8137.2002.00367.x

[B55] Lilleskov E. A. Hobbie E. A. Horton T. R. (2011). Conservation of ectomycorrhizal fungi: exploring the linkages between functional and taxonomic responses to anthropogenic N deposition. *Fungal Ecol.* 4 174–183. 10.1016/j.funeco.2010.09.008

[B56] Liu Y. Zuo S. Xu L. Zou Y. Song W. (2012). Study on diversity of endophytic bacterial communities in seeds of hybrid maize and their parental lines. *Arch. Microbiol.* 194 1001–1012. 10.1007/s00203-012-0836-838 22892578

[B57] Marasco R. Rolli E. Ettoumi B. Vigani G. Mapelli F. Borin S. (2012). A drought resistance-promoting microbiome is selected by root system under desert farming. *PLoS ONE* 7:e48479 10.1371/journal.pone.0048479 PMC3485337 23119032

[B58] McCune B. Grace J. B. (2002). *Analysis of Ecological Communities.* Gleneden Beach, OR: MjM Software Design.

[B59] McMurdie P. J. Holmes S. (2013). phyloseq: an R package for reproducible interactive analysis and graphics of microbiome census data. *PLoS ONE* 8:e61217 10.1371/journal.pone.0061217 PMC3632530 23630581

[B60] Moeller H. V. Peay K. G. Fukami T. (2014). Ectomycorrhizal fungal traits reflect environmental conditions along a coastal California edaphic gradient. *Fems Microbiol. Ecol.* 87 797–806. 10.1111/1574-6941.12265 24289145

[B61] Naveed M. Mitter B. Reichenauer T. G. Wieczorek K. Sessitsch A. (2014). Increased drought stress resilience of maize through endophytic colonization by Burkholderia phytofirmans PsJN and *Enterobacter* sp. FD17. *Environ. Exp. Bot.* 97 30–39. 10.1016/j.envexpbot.2013.09.014

[B62] O’Brien H. E. Parrent J. L. Jackson J. A. Moncalvo J. M. Vilgalys R. (2005). Fungal community analysis by large-scale sequencing of environmental samples. *Appl. Environ. Microbiol.* 71 5544–5550. 10.1128/AEM.71.9.5544-5550.2005 16151147 PMC1214672

[B63] Oksanen J. Blanchet F. G. Kindt R. Legendre P. Minchin P. R. O’Hara R. B. (2015). *vegan: ommunity Ecology Package*. R package version 2.3-0 Available at: http://CRAN.R-project.org/package=vegan

[B64] Oono R. Lutzoni F. Arnold A. E. Kaye L. U’Ren J. M. May G. (2014). Genetic variation in horizontally transmitted fungal endophytes of pine needles reveals population structure in cryptic species. *Am. J. Bot.* 101 1362–1374. 10.3732/ajb.1400141 25156984

[B65] Pankievicz V. C. S. do Amaral F. P. Santos K. F. D. N. Agtuca B. Xu Y. Schueller M. J. (2015). Robust biological nitrogen fixation in a model grass–bacterial association. *Plant J.* 81 907–919. 10.1111/tpj.12777 25645593

[B66] Peay K. G. Kennedy P. G. Bruns T. D. (2008). Fungal community ecology: a hybrid beast with a molecular master. *Bioscience* 58 799–810. 10.1641/B580907

[B67] Pianka E. R. (1981). “Competition and niche theory,” in *Theoretical ecology principles and applications*, ed. May R. M. (Hoboken, NJ: Blackwell Scientific), 167–196.

[B68] Pirttilä A. M. Joensuu P. Pospiech H. Jalonen J. Hohtola A. (2004). Bud endophytes of Scots pine produce adenine derivatives and other compounds that affect morphology and mitigate browning of callus cultures. *Physiol. Plant.* 121 305–312. 10.1111/j.0031-9317.2004.00330.x 15153198

[B69] Pirttilä A. M. Laukkanen H. Pospiech H. Myllylä R. Hohtola A. (2000). Detection of Intracellular Bacteria in the Buds of Scotch Pine (*Pinus sylvestris* L.) by In Situ Hybridization. *Appl. Environ. Microbiol.* 66 3073–3077. 10.1128/aem.66.7.3073-3077.2000 10877808 PMC92113

[B70] Pirttilä A. M. Pospiech H. Laukkanen H. Myllylä R. Hohtola A. (2005). Seasonal variations in location and population structure of endophytes in buds of Scots pine. *Tree Physiol.* 25 289–297. 10.1093/treephys/25.3.289 15631977

[B71] Pirttilä A. M. Wäli P. (2009). “Conifer endophytes,” in *Defensive Mutualism in Microbial Symbiosis*, eds White J. F. Jr. Torres M. S. (Boca Raton, FL: CRC Press), 235–246. 10.1201/9781420069327.ch15

[B72] Pohjanen J. Koskimäki J. J. Sutela S. Ardanov P. Suorsa M. Niemi K. (2014). Interaction with ectomycorrhizal fungi and endophytic Methylobacterium affects nutrient uptake and growth of pine seedlings in vitro. *Tree Physiol.* 34 993–1005. 10.1093/treephys/tpu062 25149086

[B73] Poole A. M. Stouffer D. B. Tylianakis J. M. (2012). ‘Ecosystomics’: ecology by sequencer. *Trends Ecol. Evol.* 27 309–310. 10.1016/j.tree.2012.03.008 22537669

[B74] Price M. N. Dehal P. S. Arkin A. P. (2009). FastTree: computing large minimum evolution trees with profiles instead of a distance matrix. *Mol. Biol. Evol.* 26 1641–1650. 10.1093/molbev/msp077 19377059 PMC2693737

[B75] R Core Team (2015). *R: A Language and Environment for Statistical Computing*. Vienna: R Foundation for Statistical Computing Available at: http://www.R-project.org/

[B76] Rastogi G. Sbodio A. Tech J. J. Suslow T. V. Coaker G. L. Leveau J. H. J. (2012). Leaf microbiota in an agroecosystem: spatiotemporal variation in bacterial community composition on field-grown lettuce. *ISME J.* 6 1812–1822. 10.1038/ismej.2012.32 22534606 PMC3446804

[B77] Redford A. J. Bowers R. M. Knight R. Linhart Y. Fierer N. (2010). The ecology of the phyllosphere: geographic and phylogenetic variability in the distribution of bacteria on tree leaves. *Environ. Microbiol.* 12 2885–2893. 10.1111/j.1462-2920.2010.02258.x 20545741 PMC3156554

[B78] Rhim S.-L. Völksch B. Gardan L. Paulin J.-P. Langlotz C. Kim W.-S. (1999). *Erwinia pyrifoliae*, an Erwinia species different from *Erwinia amylovora*, causes a necrotic disease of Asian pear trees. *Plant Pathol.* 48 514–520. 10.1046/j.1365-3059.1999.00376.x

[B79] Richardson D. M. (ed.). (2000). *Ecology and Biogeography of Pinus.* Cambridge: Cambridge University Press.

[B80] Rillig M. C. Antonovics J. Caruso T. Lehmann A. Powell J. R. Veresoglou S. D. (2015). Interchange of entire communities: microbial community coalescence. *Trends Ecol. Evol.* 30 470–476. 10.1016/j.tree.2015.06.004 26111582

[B81] Rúa M. Moore B. Hergott N. Van L. Jackson C. Hoeksema J. (2015). Ectomycorrhizal fungal communities and enzymatic activities vary across an ecotone between a forest and field. *J. Fungi* 1 185–210. 10.3390/jof1020185 PMC5753110 29376908

[B82] Rúa M. A. Umbanhowar J. Hu S. Burkey K. O. Mitchell C. E. (2013). Elevated CO_2_ spurs reciprocal positive effects between a plant virus and an arbuscular mycorrhizal fungus. *New Phytol.* 199 541–549. 10.1111/nph.12273 23594373

[B83] Ryberg M. Matheny P. B. (2012). Asynchronous origins of ectomycorrhizal clades of Agaricales. *Proc. R. Soc. B Biol. Sci.* 279 2003–2011. 10.1098/rspb.2011.2428 PMC3311903 22171078

[B84] Schardl C. L. Phillips T. D. (1997). Protective Grass Endophytes: where are they from and where are they going? *Plant Disease* 81 430–438. 10.1094/PDIS.1997.81.5.430 30861917

[B85] Selosse M.-A. Richard F. He X. Simard S. W. (2006). Mycorrhizal networks: des liaisons dangereuses? *Trends Ecol. Evol.* 21 621–628. 10.1016/j.tree.2006.07.003 16843567

[B86] Sevilla M. Burris R. H. Gunapala N. Kennedy C. (2001). Comparison of benefit to sugarcane plant growth and 15N2 incorporation following inoculation of sterile plants with acetobacter diazotrophicus wild-type and nif- mutant strains. *Mol. Plant Microbe Interact.* 14 358–366. 10.1094/MPMI.2001.14.3.358. 11277433

[B87] Shen S. Y. Fulthorpe R. (2015). Seasonal variation of bacterial endophytes in urban trees. *Front. Microbiol.* 6:427 10.3389/fmicb.2015.00427 PMC4437045 26042095

[B88] Shrestha R. Koo J.-H. Park D.-H. Hwang I.-G. Hur J.-H. Lim C.-K. (2003). Erwinia pyrifoliae, a causal endemic pathogen of shoot blight of Asian pear tree in Korea. *Plant Pathol. J.* 19 294–300. 10.5423/PPJ.2003.19.6.294

[B89] Simpson G. L. (2015). *ggvegan: ‘ggplot2’ Plots for the ‘vegan’ Package*. R package version 0 0–3.

[B90] Six J. Frey S. D. Thiet R. K. Batten K. M. (2006). Bacterial and fungal contributions to carbon sequestration in agroecosystems. *Soil Sci. Soc. Am. J.* 70 555–569. 10.2136/sssaj2004.0347

[B91] Smith M. D. Douhan G. Rizzo D. (2007). Ectomycorrhizal community structure in a xeric Quercus woodland based on rDNA sequence analyzsis of sporocarps and pooled roots. *New Phytol.* 174 847–863. 10.1111/j.1469-8137.2007.02040.x 17504467

[B92] Smith S. E. Read D. (2008). *Mycorrhizal Symbiosis.* London: Elsevier.

[B93] Suz L. M. Barsoum N. Benham S. Dietrich H.-P. Fetzer K. D. Fischer R. (2014). Environmental drivers of ectomycorrhizal communities in Europe’s temperate oak forests. *Mol. Ecol.* 23 5628–5644. 10.1111/mec.12947 25277863

[B94] Tedersoo L. May T. W. Smith M. E. (2010). Ectomycorrhizal lifestyle in fungi: global diversity, distribution, and evolution of phylogenetic lineages. *Mycorrhiza* 20 217–263. 10.1007/s00572-009-0274-x 20191371

[B95] Upreti R. Thomas P. (2015). Root-associated bacterial endophytes from *Ralstonia solanacearum* resistant and susceptible tomato cultivars and their pathogen antagonistic effects. *Front. Microbiol.* 6:255 10.3389/fmicb.2015.00255 PMC4396348 25926818

[B96] Van Der Heijden M. G. A. Bardgett R. D. Van Straalen N. M. (2008). The unseen majority: soil microbes as drivers of plant diversity and productivity in terrestrial ecosystems. *Ecol. Lett.* 11 296–310. 10.1111/j.1461-0248.2007.01139.x 18047587

[B97] Van Der Putten W. H. (2009). A multitrophic perspective on functioning and evolution of facilitation in plant communities. *J. Ecol.* 97 1131–1138. 10.1111/j.1365-2745.2009.01561.x

[B98] van der Putten W. H. Bardgett R. D. Bever J. D. Bezemer T. M. Casper B. B. Fukami T. (2013). Plant–soil feedbacks: the past, the present and future challenges. *J. Ecol.* 101 265–276. 10.1111/1365-2745.12054

[B99] Vetriani C. Crespo-Medina M. Antunes A. (2014). “The family salinisphaeraceae,” in *The Prokaryotes – Gammaproteobacteria*, eds Rosenberg E. Delong E. F. Lory S. Stackebrandt E. Thompson F. (Berlin: Springer-Verlag), 591–596.

[B100] Vu V. Q. (2011). *ggbiplot: A ggplot2 Based Biplot*. R package version 0.55 Available at: http://github.com/vqv/ggbiplot

[B101] Wagg C. Jansa J. Schmid B. van der Heijden M. G. A. (2011). Belowground biodiversity effects of plant symbionts support aboveground productivity. *Ecol. Lett.* 14 1001–1009. 10.1111/j.1461-0248.2011.01666.x 21790936

[B102] Wardle D. A. Bardgett R. D. Klironomos J. N. Setala H. van der Putten W. H. Wall D. H. (2004). Ecological linkages between aboveground and belowground biota. *Science* 304 1629–1633. 10.1126/science.1094875 15192218

[B103] Wickham H. (2009). *GGplot2: Elegant Graphics for Data Analysis.* New York, NY: Springer.

