# Supplementary material for: Associations between Ectomycorrhizal Fungi and Bacterial Needle Endophytes in Pinus radiata: Implications for Biotic Selection of Microbial Communities
Source: Front Microbiol. 2016 Mar 31;7:399. doi: 10.3389/fmicb.2016.00399 (PMC4815291; doi:10.3389/fmicb.2016.00399)

Supplementary Material

Associations between ectomycorrhizal fungi and bacterial needle endophytes in *Pinus radiata*: implications for biotic selection of microbial communities

Megan A. Rúa*, Emily C. Wilson, Sarah Steele, Arielle R. Munters, Jason D. Hoeksema, Anna C. Frank

*** Correspondence:** Megan A. Rúa, National Institute for Mathematical and Biological Synthesis, University of Tennessee, 1122 Volunteer Blvd., Knoxville, TN, 37996-3410, USA.

Supplementary Material

Environmental Characteristics

Supplementary Figure 1. Soil characteristics for samples taken underneath Monterey pine (*Pinus radiata*) across three populations in northern California, USA. Values represent the mean ± standard error for % silt (A), % clay (B), % sand (C), soil water content (D), and % sand : % silt (E). Letters indicate differences by population based on Tukey's HSD for each soil characteristic.

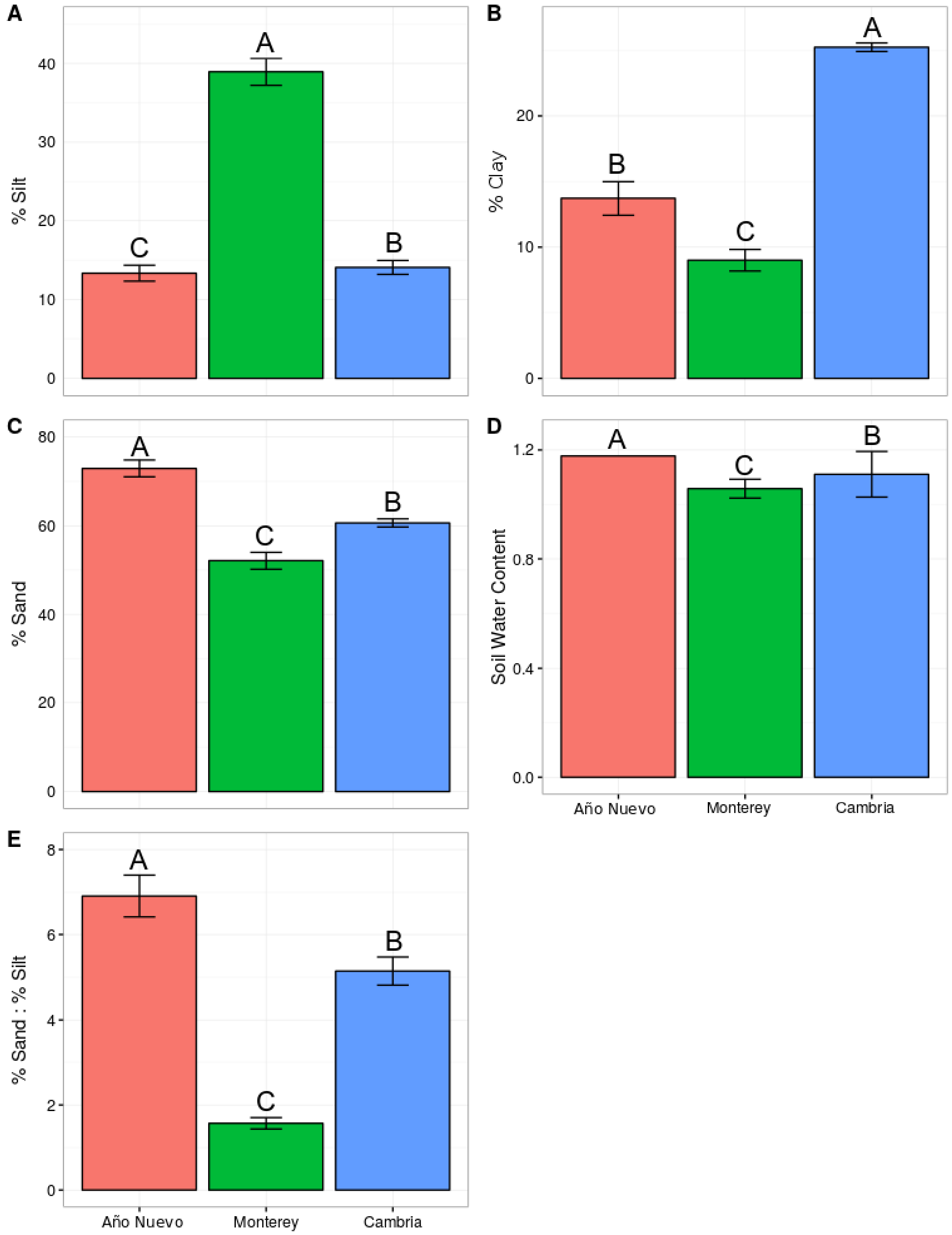

Supplementary Figure 2. Environmental factor PCA. Ellipses indicate 95% confidence intervals for samples collected from Año Nuevo (salmon), Monterey (green), and Cambria (blue). Arrows specify association of environmental variables with particular sites.

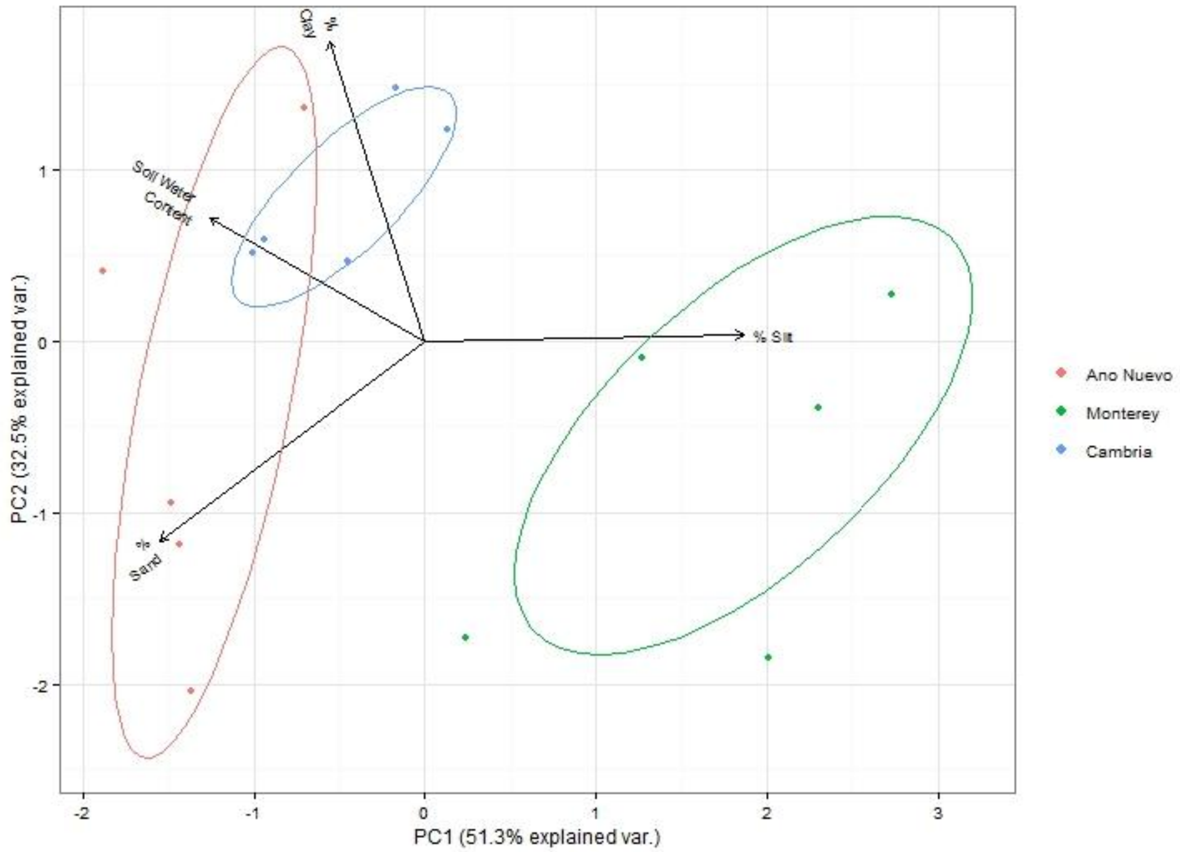

Bacterial Endophytes

Supplementary Figure 3. Bacterial rarefaction plot.

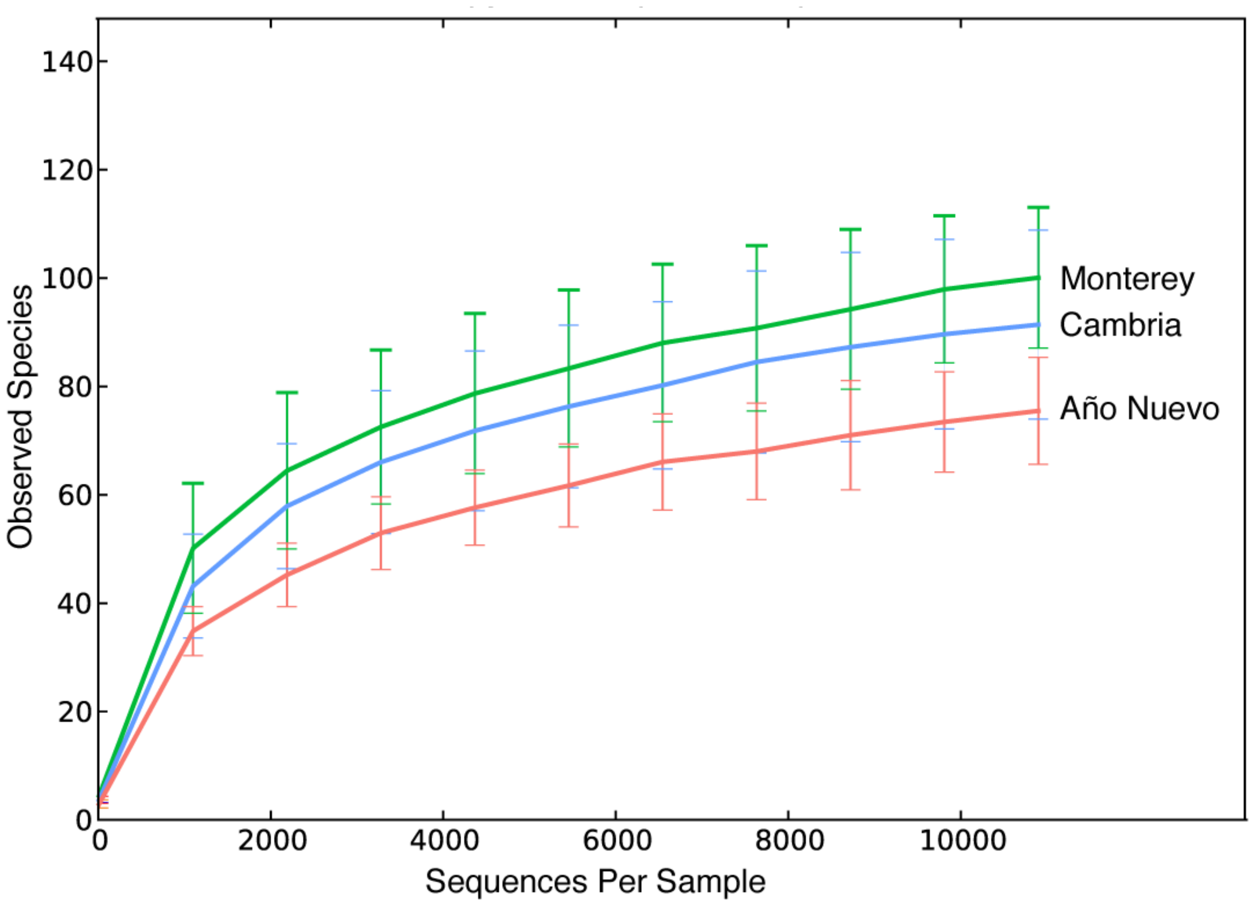

Supplementary Figure 4. Alpha diversity scatterplot (by site) for A-C: bacteria and D-F: fungi. Alpha diversity was assessed as observed values (A,D), Chao1 (B,E), and the Shannon index (C,F).

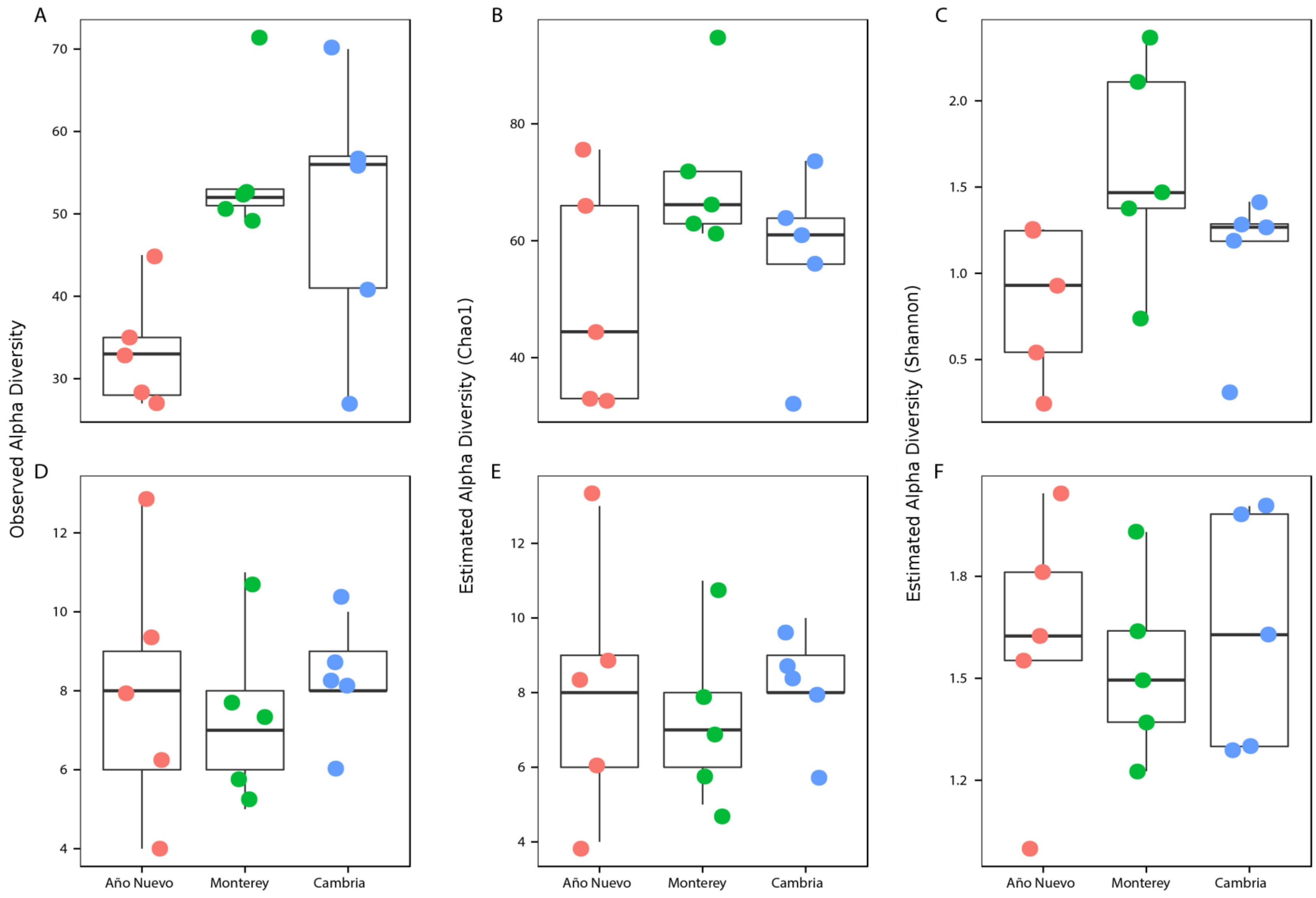

Supplementary Figure 5. Alpha diversity by environment for bacteria when environmental characteristics are assessed separately: soil water (A-C), % silt (D-F), % sand (G-I), and % clay (J-L). Alpha diversity was assessed as observed values (A,D,G,J), Chao1 (B,E,H,K), and the Shannon index (C,F,I,L).

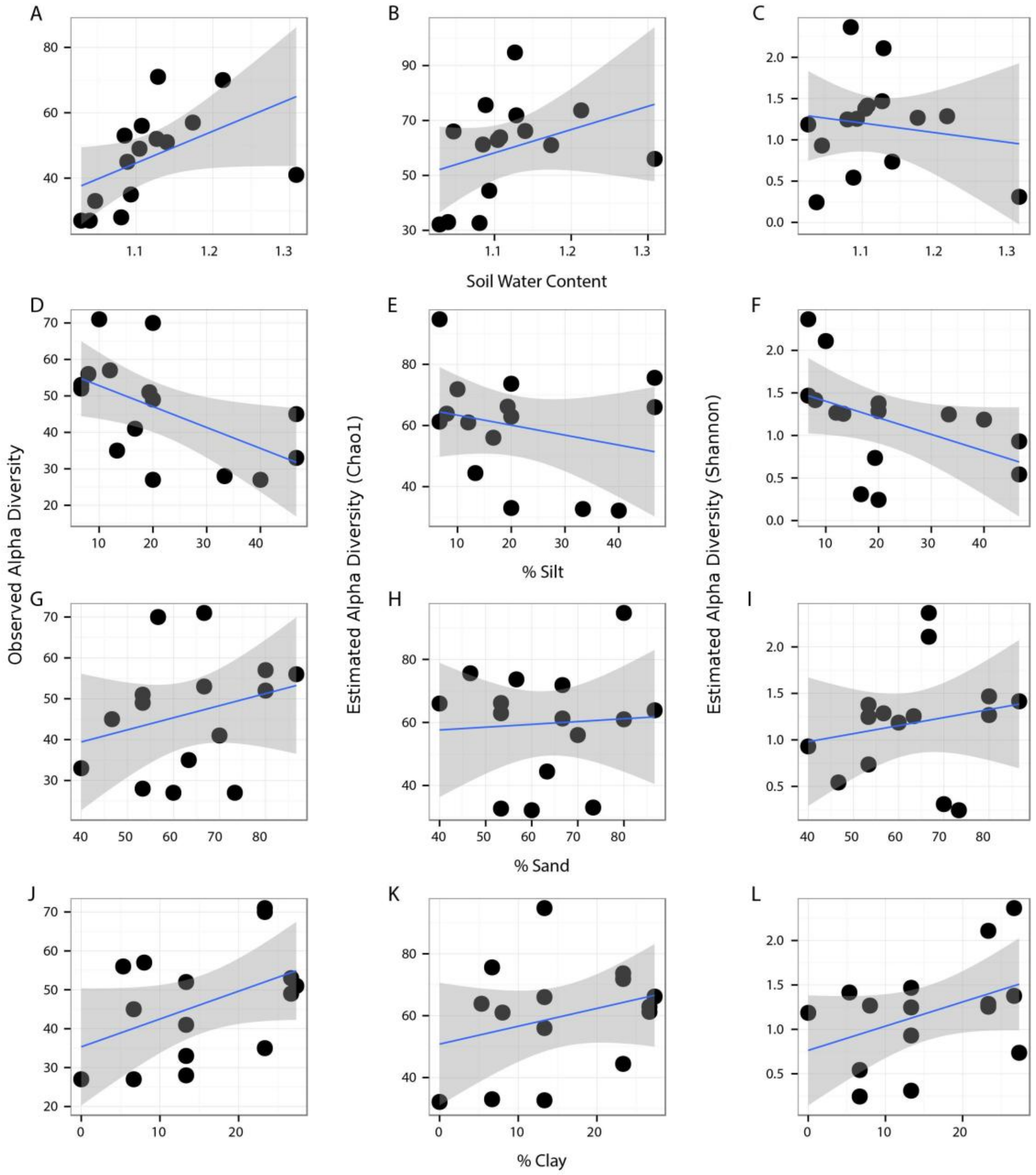

Supplementary Figure 6. Alpha diversity by environment for bacteria when % sand and percent silt are assessed as a single metric: soil water (A-C), % sand : % silt (D-F), and % clay (G-I). Alpha diversity was assessed as observed values (A,D,G), Chao1 (B,E,H), and the Shannon index (C,F,I). When % silt and % sand were combined into a single metric, neither soil water content ($F_{1,11}$=1.894, p=0.1961), nor any of the measures of soil texture (% sand:silt: $F_{1,11}$= 2.325, p= 0.1555, % clay: $F_{1,11}$= 0.6806, p= 0.4269) significantly altered alpha diversity as assessed by the Chao1 index (B,E,H). When alpha diversity was assessed using the Shannon index, % sand:silt significantly increased alpha diversity ($F_{1,11}$= 8.609, p= 0.0136), but soil water content ($F_{1,11}$= 0.5654, p= 0.4679) and % clay ($F_{1,11}$= 5.2824, p= 0.0522) did not significantly alter alpha diversity (C,F,I).

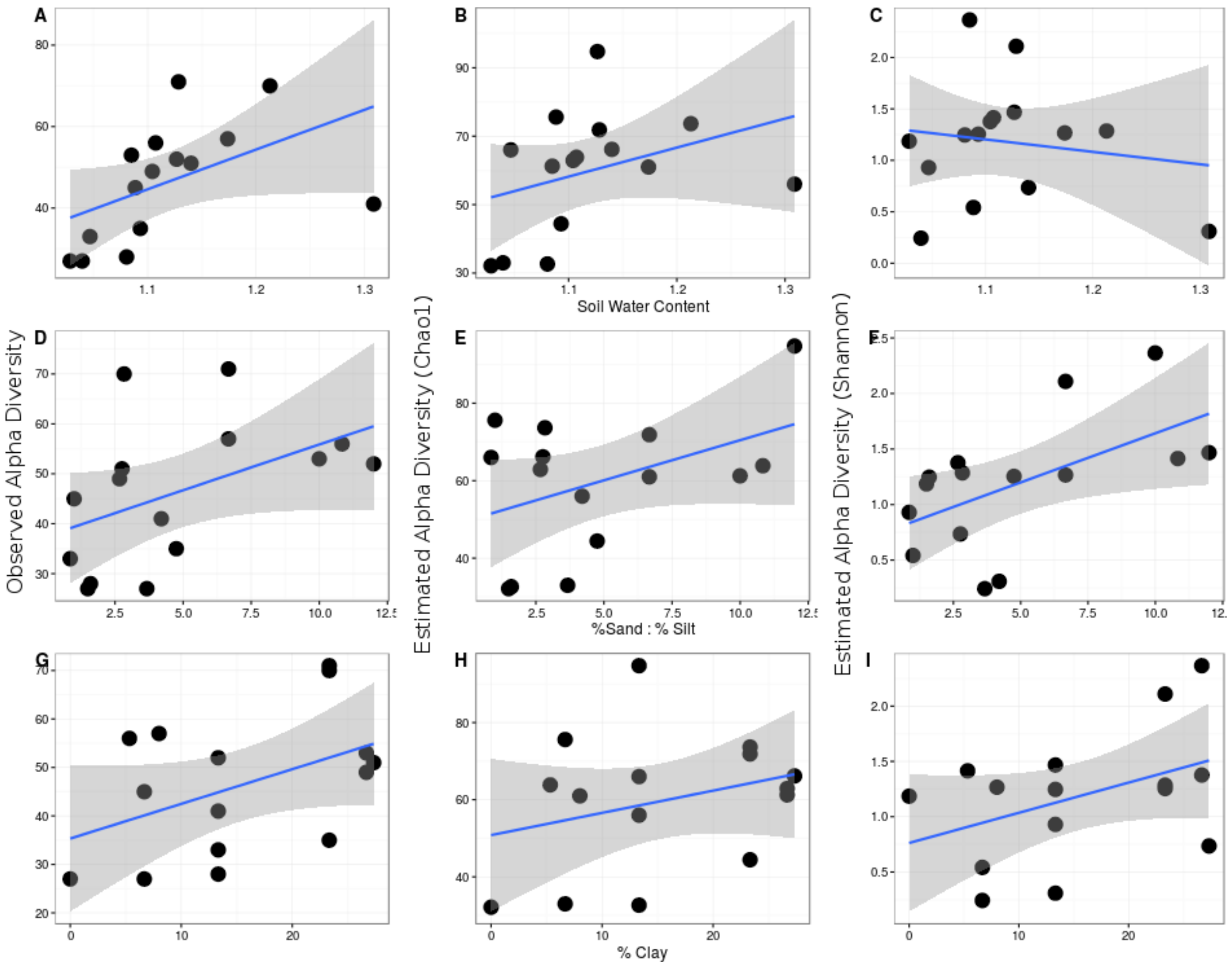

Supplementary Figure 7. Nonmetric multidimensional scaling ordinations for bacteria as a function of environmental variables. Ellipses indicate 95% confidence intervals for samples collected from Año Nuevo (salmon), Monterey (green), and Cambria (blue). Arrows specify association of environmental variables with particular sites.

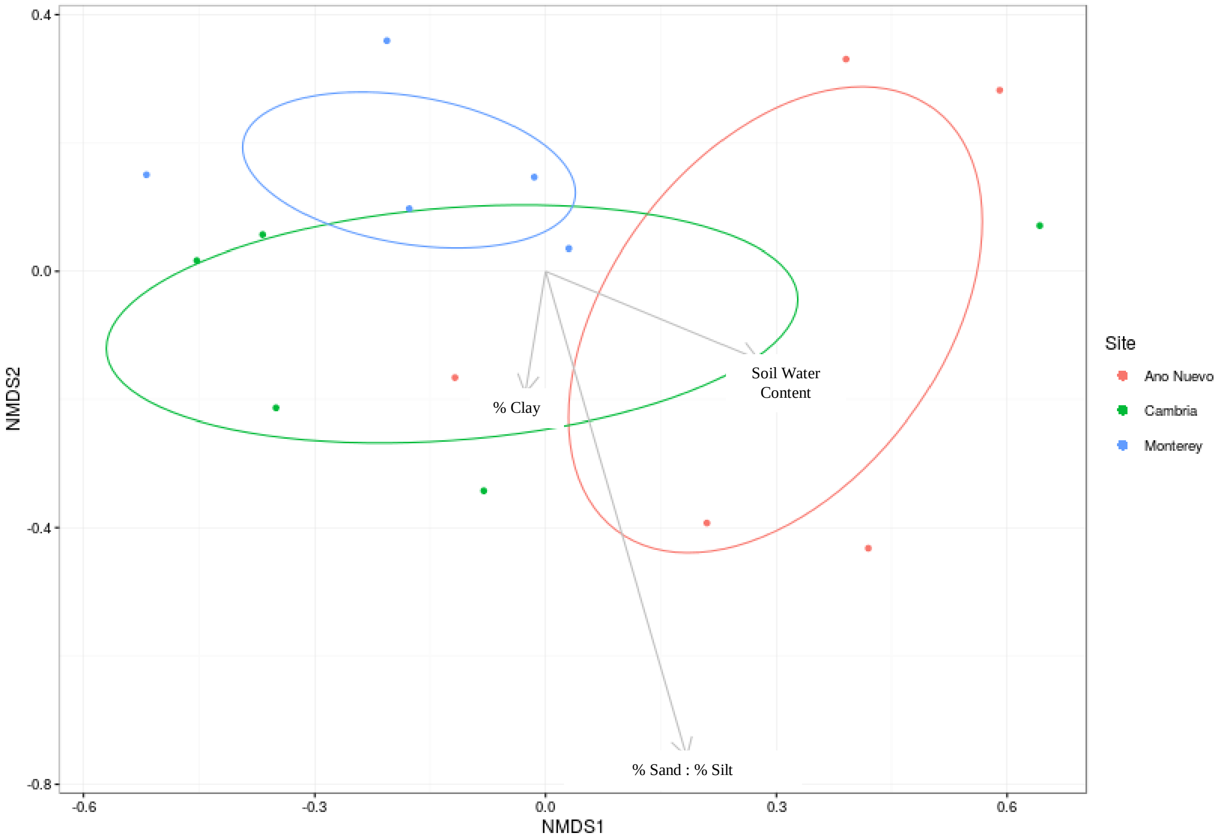

Supplementary Figure 8. Heatmap relating environmental characteristics with bacterial OTUs.

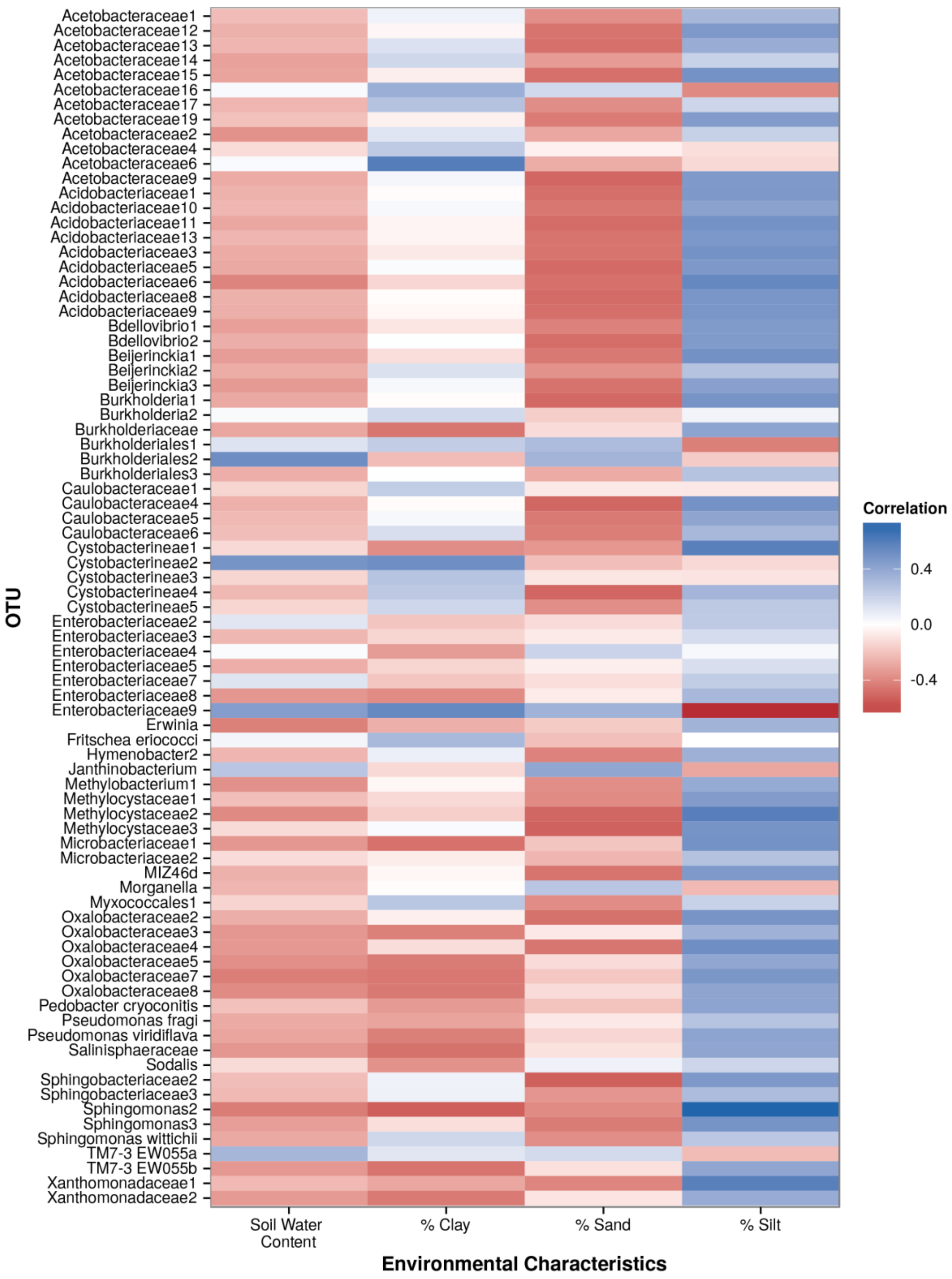

Supplementary Figure 9. Heatmap relating environmental characteristics with bacterial OTUs.

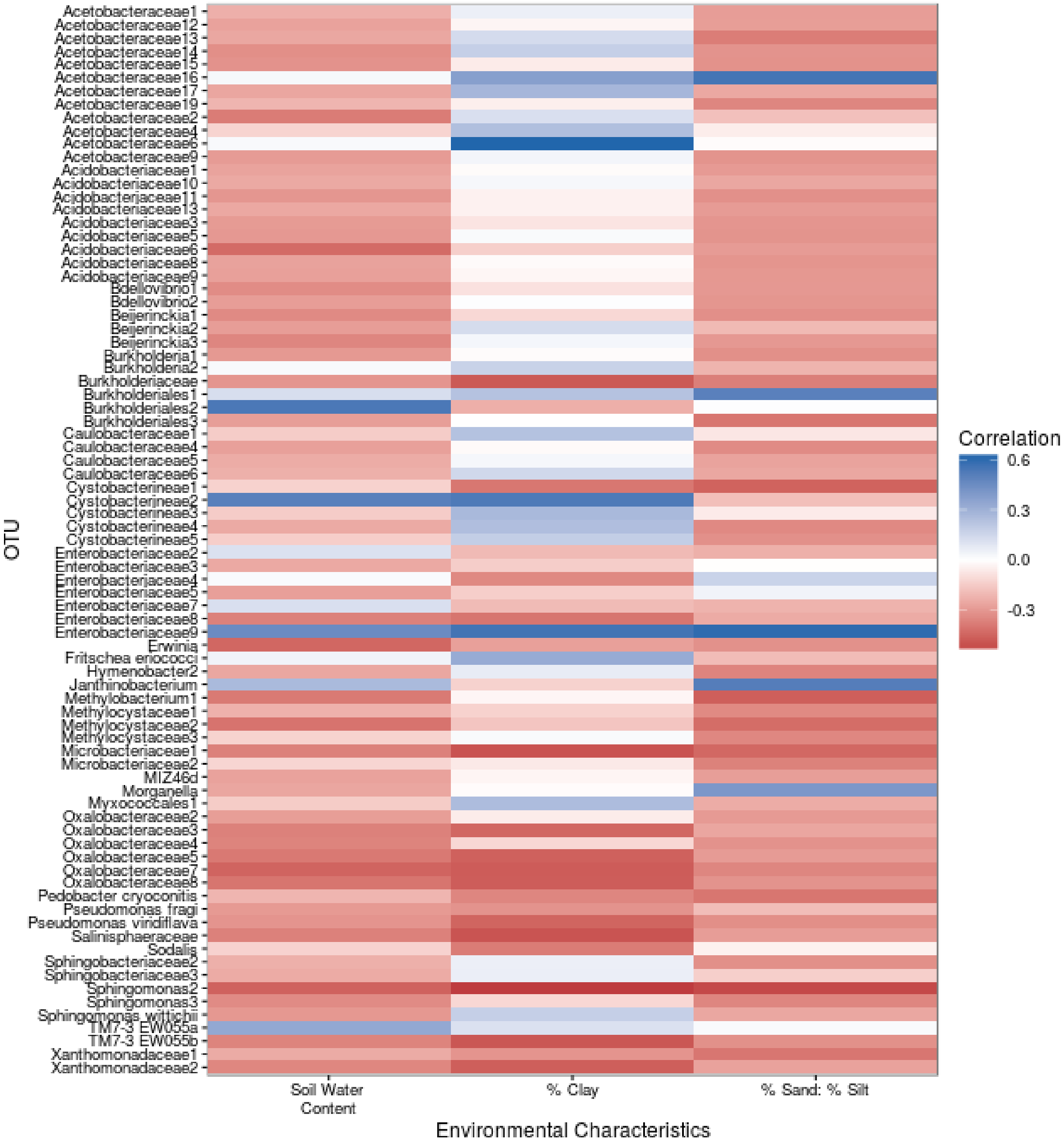

Supplementary Figure 10. Alpha diversity scatterplot by site and tissue.

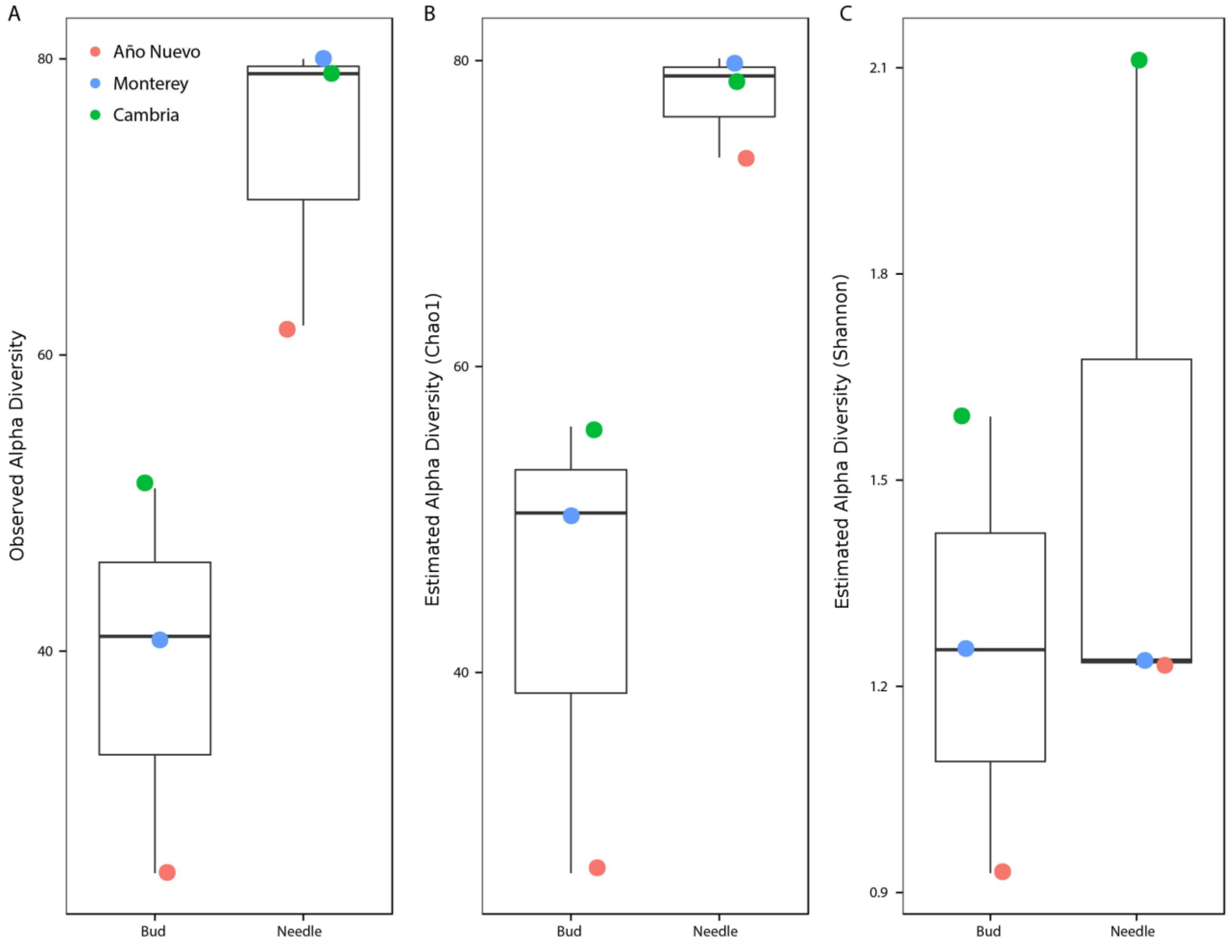

Supplementary Figure 11. Nonmetric multidimensional scaling ordinations for bacteria by tissue type.

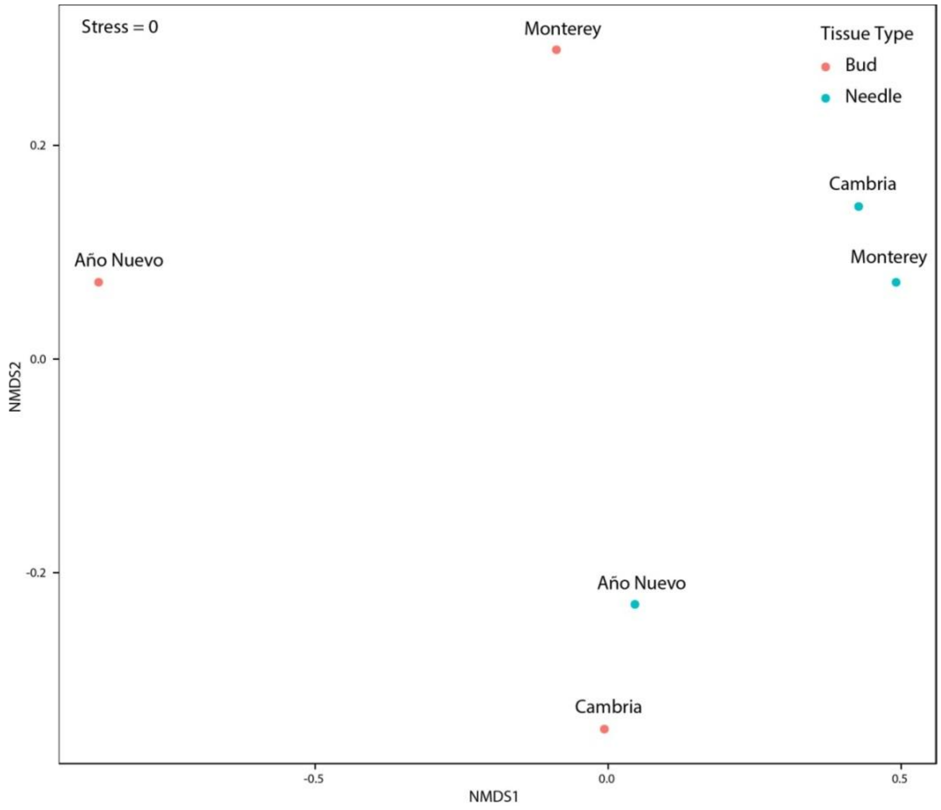

ECM Fungal Species

Supplementary Figure 12. Alpha diversity by environment for fungi when environmental characteristics are assessed separately: soil water (A-C), % silt (D-F), % sand (G-I), and % clay (J-L). Alpha diversity was assessed as observed values (A,D,G,J), Chao1 (B,E,H,K), and the Shannon index (C,F,I,L).

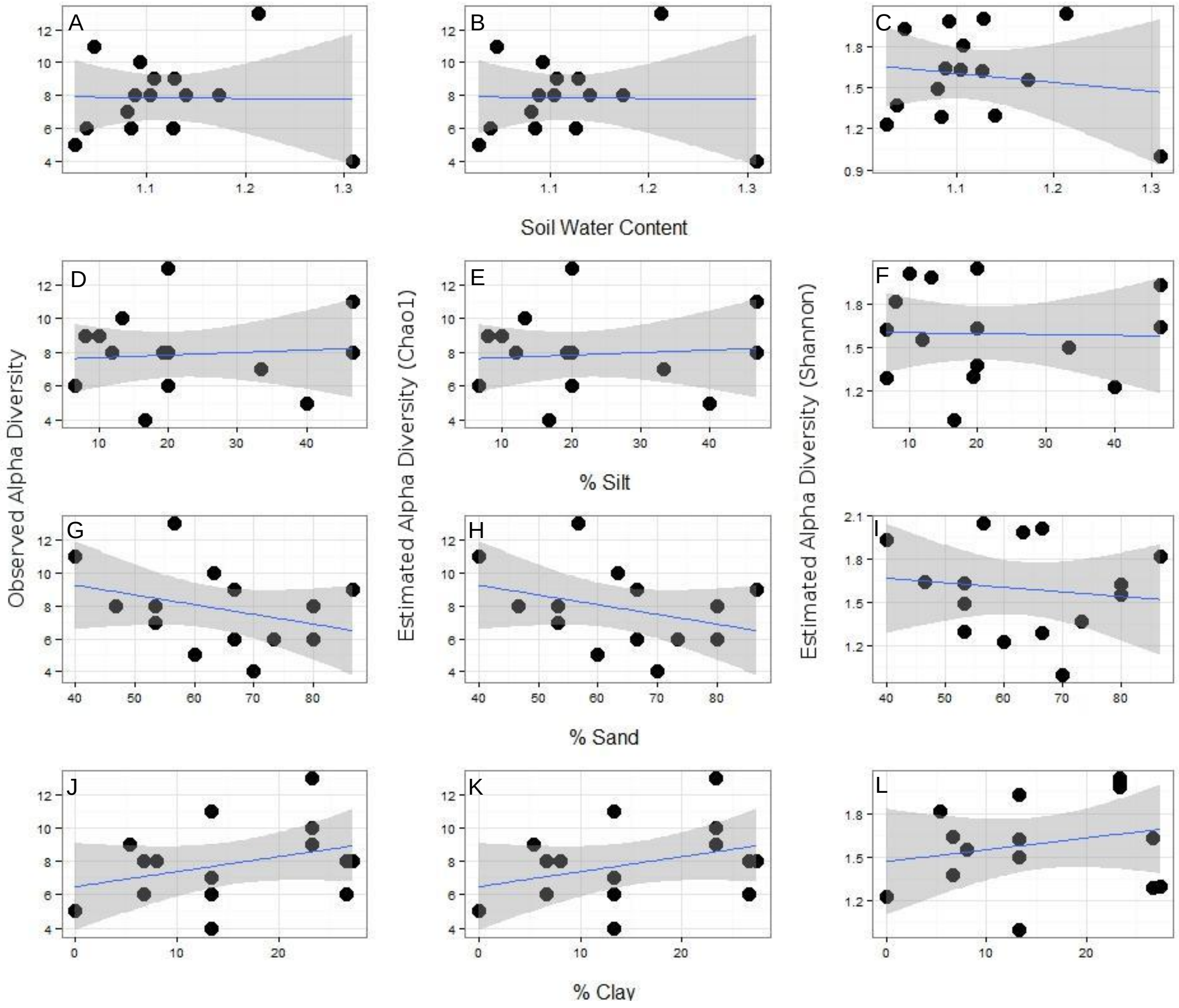

Supplementary Figure 13. Alpha diversity by environment for ECM fungi when % sand and % silt are assessed as a single metric: soil water (A-C), % sand : % silt (D-F), and % clay (G-I). Alpha diversity was assessed as observed values (A,D,G), Chao1 (B,E,H), and the Shannon index (C,F,I). When % silt and % sand were combined into a single metric, no difference in alpha diversity was found among environmental characteristics for either the Chao1 index (soil water content: $F_{1,11}$=0.0039, p=0.9515; % sand : % silt: $F_{1,11}$=0.3894, p=0.5453; % clay: $F_{1,11}$=1.739, p=0.214) or the Shannon index (soil water content: $F_{1,11}$=0.2787, p=0.6081; % sand : % silt: $F_{1,11}$=0.0746, p=0.7898; % clay: $F_{1,11}$=0.9205, p=0.358).

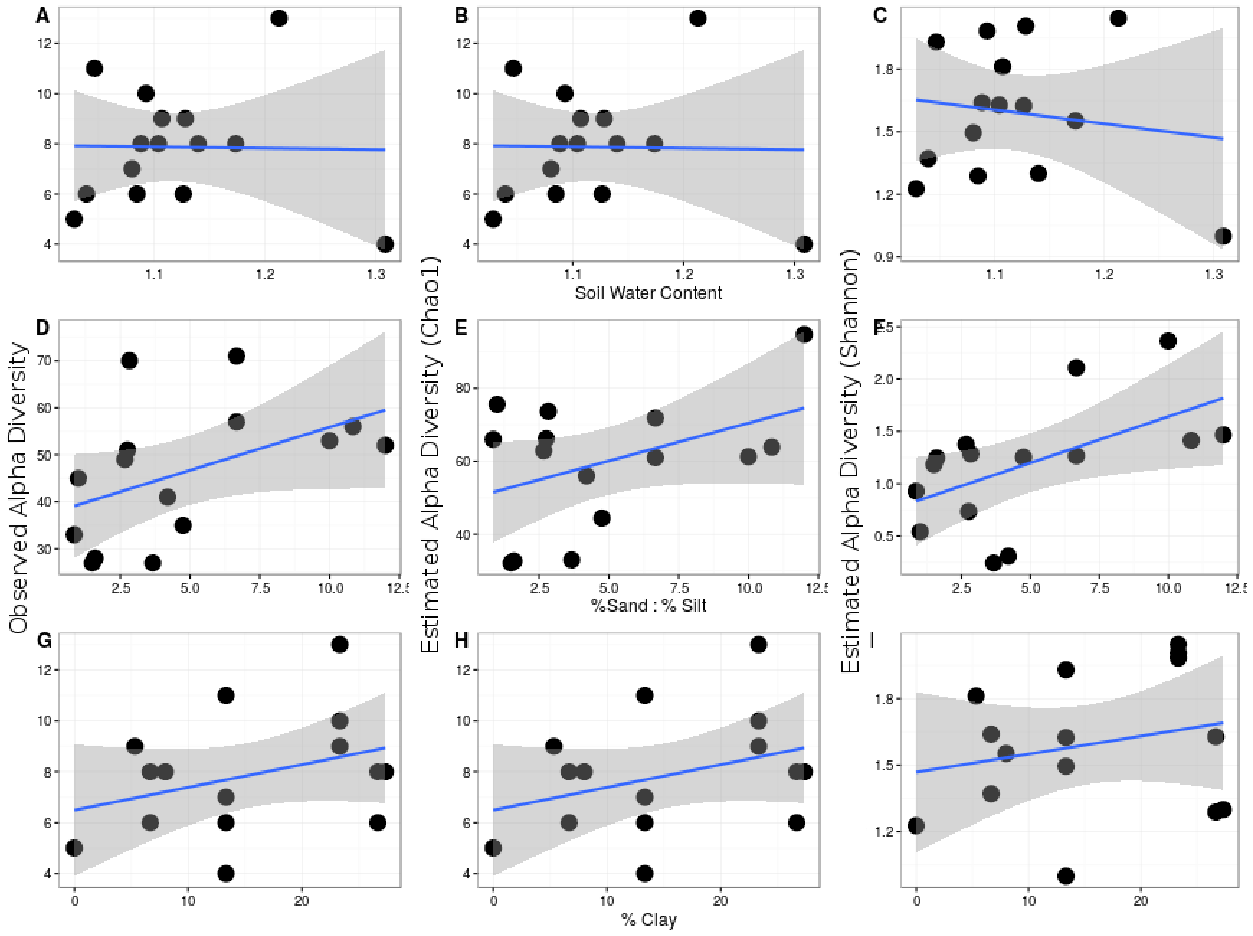

Supplementary Figure 14. Nonmetric multidimensional scaling ordinations for fungi as a function of environmental variables. Ellipses indicate 95% confidence intervals for samples collected from Año Nuevo (salmon), Monterey (green), and Cambria (blue). Arrows specify association of environmental variables with particular sites.Soil water content was a significant structuring factor of mycorrhizal assemblages (R^2=0.09, p=0.044) but none of the measures of soil texture (% sand: % silt: R^2=0.07, p=0.62, % clay: R^2=0.07, p=0.316) were significant structuring factors.

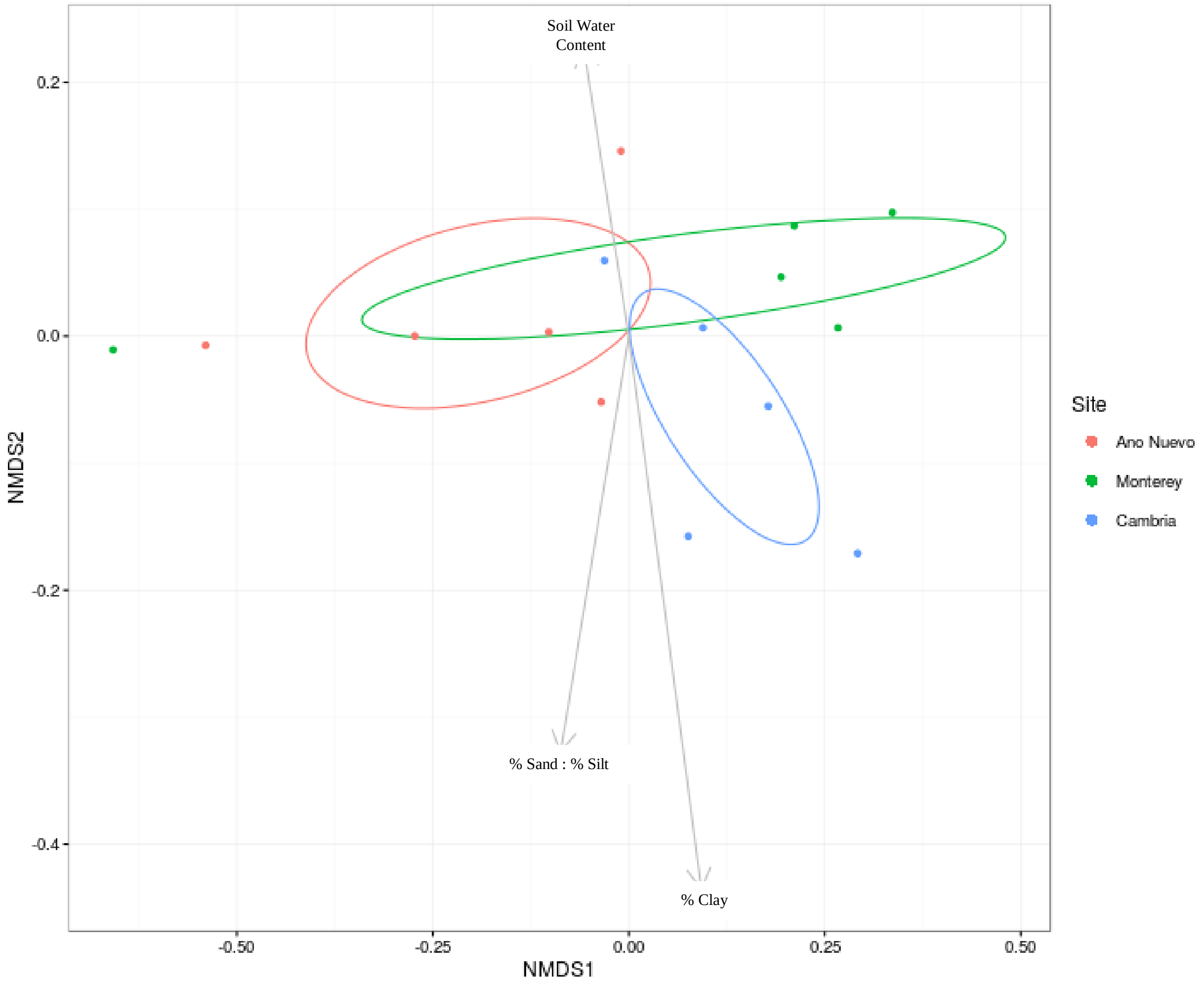

Supplementary Figure 15. Heatmap relating environmental characteristics with ECM fungal OTUs.

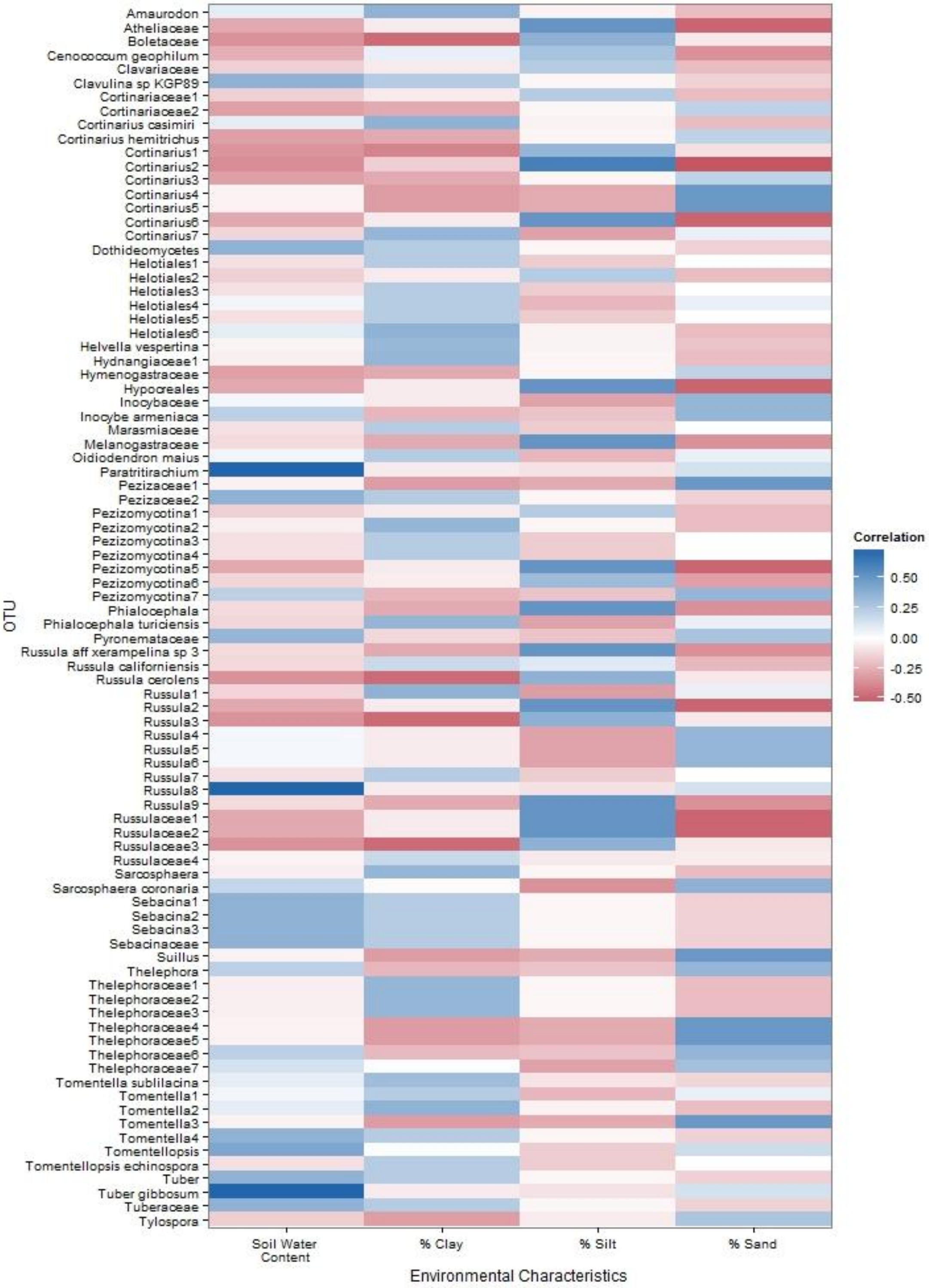

Supplementary Figure 16. Heatmap relating environmental characteristics with ECM fungal OTUs.

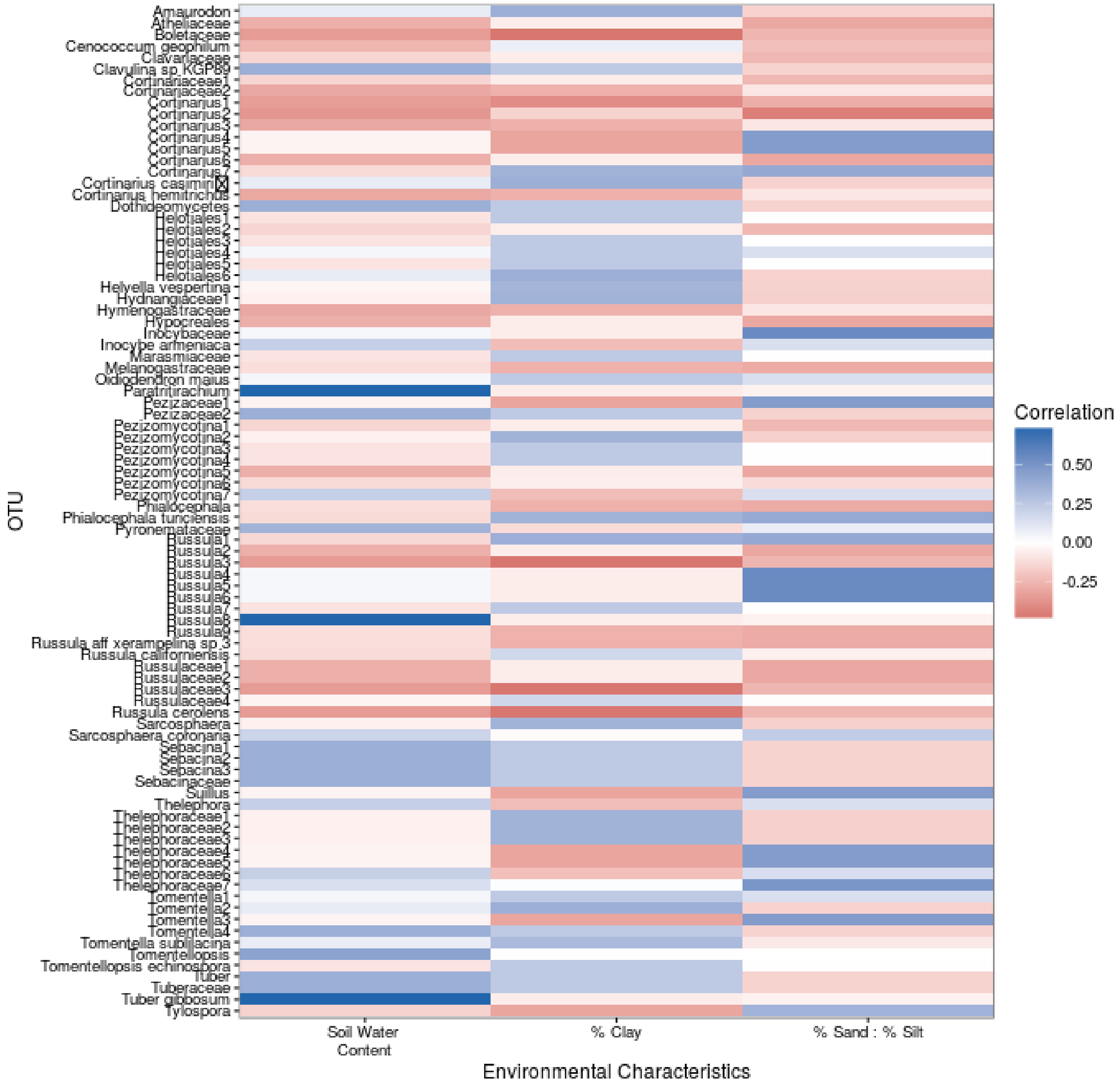

Supplementary Figure 17. Fungal trait distribution by site.

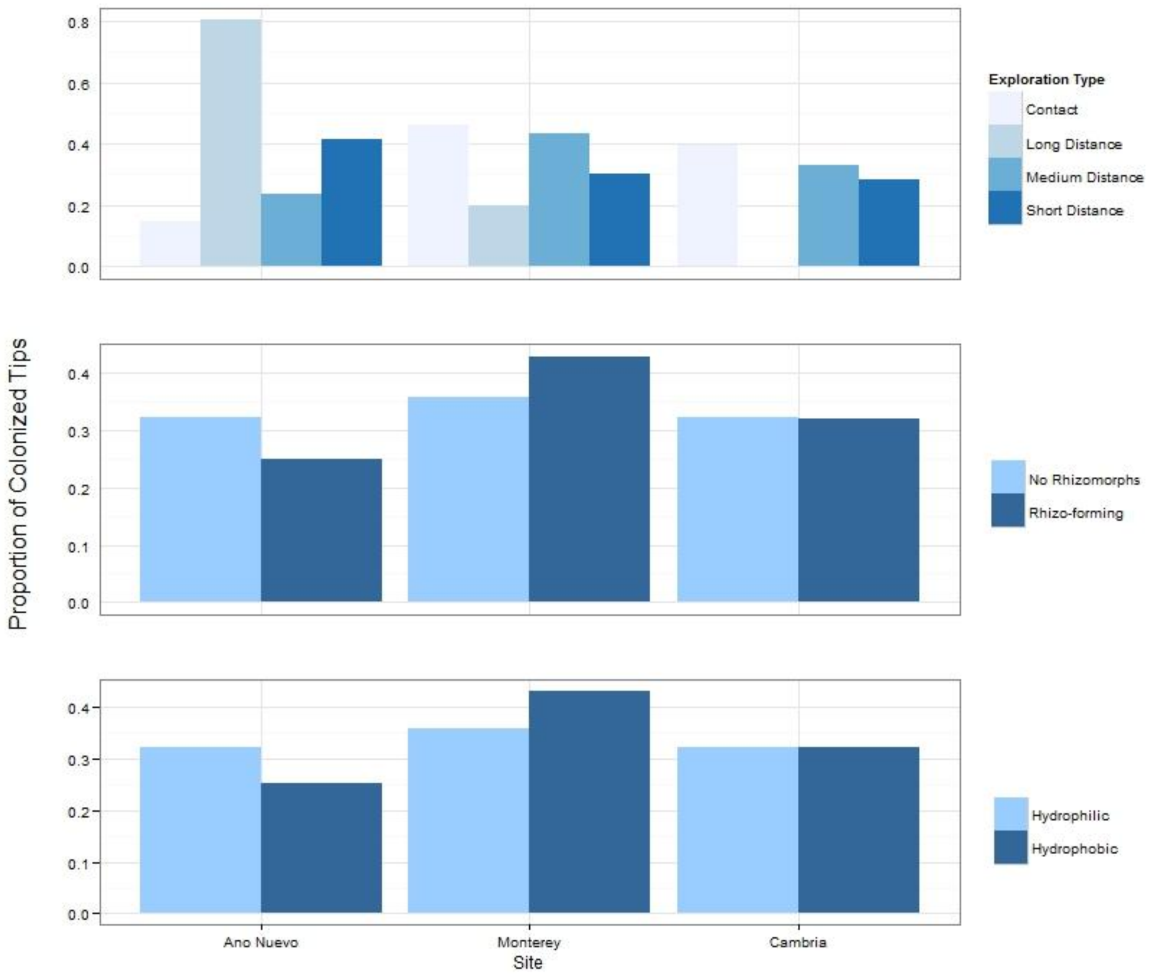

Supplementary Figure 18. Exploration type constrained analysis of principal coordinates with no conditions.

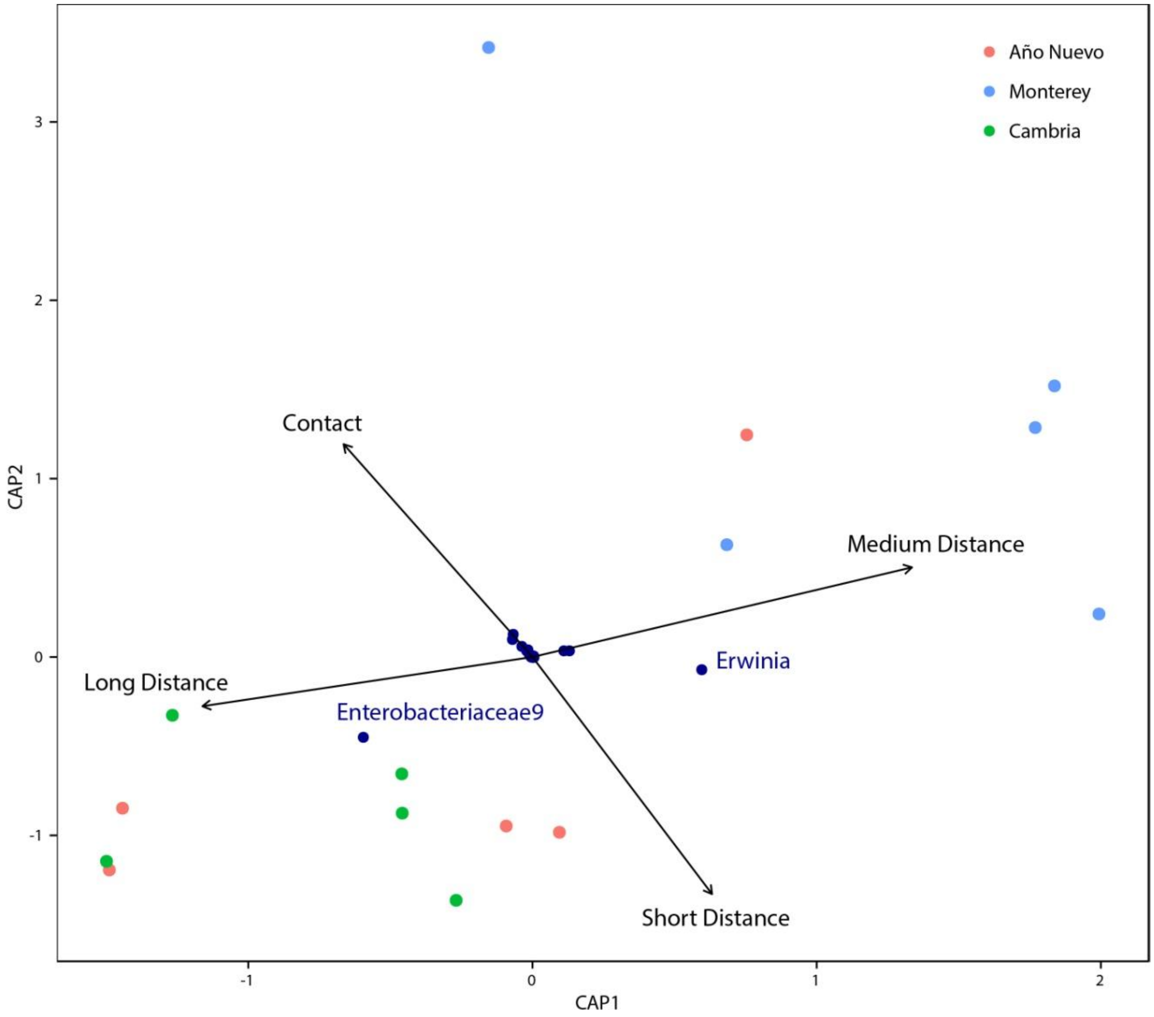

Supplement: Supplementary file 1 [file Data_Sheet_1.PDF]
